# Next-Generation Phylogeography: A Targeted Approach for Multilocus Sequencing of Non-Model Organisms

**DOI:** 10.1371/journal.pone.0034241

**Published:** 2012-03-28

**Authors:** Jonathan B. Puritz, Jason A. Addison, Robert J. Toonen

**Affiliations:** 1 Hawai'i Institute of Marine Biology, University of Hawai'i at Mānoa, Kāne'ohe, Hawai'i, United States of America; 2 Department of Biology, University of New Brunswick, Fredericton, New Brunswick, Canada; Lund University, Sweden

## Abstract

The field of phylogeography has long since realized the need and utility of incorporating nuclear DNA (nDNA) sequences into analyses. However, the use of nDNA sequence data, at the population level, has been hindered by technical laboratory difficulty, sequencing costs, and problematic analytical methods dealing with genotypic sequence data, especially in non-model organisms. Here, we present a method utilizing the 454 GS-FLX Titanium pyrosequencing platform with the capacity to simultaneously sequence two species of sea star (*Meridiastra calcar* and *Parvulastra exigua*) at five different nDNA loci across 16 different populations of 20 individuals each per species. We compare results from 3 populations with traditional Sanger sequencing based methods, and demonstrate that this next-generation sequencing platform is more time and cost effective and more sensitive to rare variants than Sanger based sequencing. A crucial advantage is that the high coverage of clonally amplified sequences simplifies haplotype determination, even in highly polymorphic species. This targeted next-generation approach can greatly increase the use of nDNA sequence loci in phylogeographic and population genetic studies by mitigating many of the time, cost, and analytical issues associated with highly polymorphic, diploid sequence markers.

## Introduction

Twenty years ago, phylogeography was experiencing the boom of the PCR and Sanger sequencing era [1], [2]. Rapid and cost effective sequencing of mitochondrial DNA (mtDNA) loci were driving the phylogenetic analysis of alleles and populations in a geographical context. MtDNA loci are uniparentally inherited and cannot alone represent all historical and contemporary processes acting upon a population [3], and the field as a whole recognized the need and utility of incorporating nDNA sequence data [4]–[7]. Non-coding introns are now commonly used in systematic and phylogenetic studies [8]–[10]; however, the use of nDNA sequence data, at the population level, has been hindered by technical laboratory difficulty, sequencing costs, and ambiguous analytical methods dealing with genotypic sequence data (as reviewed Hare 2001; Zhang & Hewitt 2003; Creer 2007). In turn, relatively less expensive and analytically less complex microsatellites became the overwhelming choice for phylogeography and population genetic studies of the nearly the last two decades [10]–[12].

Despite their overwhelming use in the field and utility for answering fine scale ecological questions [11], microsatellite loci, as identified by Zhang and Hewit (2003) and reviewed in [12] have two important limitations: the unclear relationship between alleles and the inconsistent and often extremely complicated modes and rates of mutation within and between loci. Phylogeographic studies specifically rely on the temporal and spatial analysis of allelic lineages to infer population processes, and any locus that relies on fragment length polymorphism has an uncertain ancestral allelic state and ambiguous genealogy [6], [13]. This uncertainty of relationships between alleles limits the utility of microsatellite data in both phylogenetic and coalescence analyses, and highlights the need for nDNA sequence data in phylogeographic studies [10].

Inferring the relationships among alleles within and between populations constitutes the primary difference between phylogeography and population genetics [2]. Theoretically, exon-primed intron crossing (EPIC) sequence loci represent an excellent nDNA marker for use in phylogeography [8]–[10], [14]. Intron sequences, while not obviously functional, are not junk DNA [15] and affect a number of eukaryotic gene expression pathways [16]–[18]. However, they are considered largely neutral [19], [20] and tend to accumulate informative mutations across sites more equally, with less homoplasy, and lower transition∶transversion ratios than coding regions [21]–[23]. Priming within the exon region allows for relatively robust primer design and enhances the ability for primers to be used across closely related taxa. Additionally, several published primer sets for “universal” EPIC loci are already widely applied and can be augmented with new primers from the abundance of published genomic data (as reviewed in [8]).

EPIC loci that are polymorphic at the population level (i.e. those that are useful for phylogeographic studies) often have relatively large amounts of genetic polymorphism due to high levels of heterozygosity and insertion-deletion (INDEL) events [8], [10]. Direct sequencing of a heterozygous locus for an INDEL polymorphism results in multiple chromatogram peeks or an apparently failed sequencing reaction [24]. Although many techniques exist to either physically separate the two sequences (cloning, single strand conformation polymorphisms, etc. [8], [10] or to empirically reconstruct the two haplotype sequences [8], [25], most approaches are costly in both time and supplies, and empirical algorithms designed to decode heterozygous chromatograms only work well with high quality data and lower levels of polymorphism, usually excluding INDELs [10], [25], [26]. In short, the extra time and money needed to incorporate highly polymorphic nuclear loci into population level studies has largely limited the use of EPIC loci in phylogeography.

Next-generation sequencing technology has the promise to overcome these obstacles to include EPIC loci in phylogeography by providing a quick and cost effective way to produce massive amounts of sequence data [27], [28]. The 454 GS-FLX titanium pyrosequencing technology (Roche Diagnostics Corporation) is particularly attractive for phylogeography with the ability to generate over 1 million reads of around 400 bp per read [29]. Moreover, these reads are obtained from the pyrosequencing of emulsion PCR (emPCR) reactions [30] which amplify DNA from only one molecule, meaning reads are the sequence of only one target molecule, the equivalent of bacterial cloning. Pyrosequencing technology can be applied to not only sequence entire genomes but can also be used to sequence pooled PCR products. When used in combination with barcoded PCR primers and a gasketed 454 sequencing plate, it enables the simultaneous sequencing of thousands of targeted loci at high coverage.

Here, we present a novel methodology utilizing the 454 sequencing platform with the capacity to simultaneously sequence two species of sea star at five different EPIC nDNA loci across 16 different populations of 20 individuals each per species. Specifically, we compare the results from three populations (three 1/16 plate libraries) with traditional Sanger sequencing based methods, and clearly demonstrate that this next-generation sequencing platform is more time and cost effective and more sensitive to rare variants than Sanger based sequencing. Moreover, the high coverage of clonally amplified sequences simplifies haplotype determination, even in highly polymorphic, non-model species. This targeted next-generation approach can greatly decrease the cost and thus increase the applications of nDNA sequence loci in phylogeographic and population genetic studies by mitigating many of the time, resource, and analytical issues associated with highly polymorphic, diploid sequence markers.

## Materials and Methods

### Overall Project Design

The use of the 454 platform to sequence 5 EPIC loci for two species of sea star was designed to complement an ongoing comparative phylogeographic study of *Meridiastra calcar* and *Parvulastra exigua* across much of their Australian ranges. The two species have a largely sympatric distribution and this method was designed to sequence 20 individuals from each species at 16 co-occurring localities. Attempts to sequence/genotype three of these loci for each species were already underway allowing for the comparisons below. From this large data set, three representative populations (one from each phylogeographic region: New South Wales, Tasmania, and Southern Australia) were chosen to compare our 454 sequencing efforts with our existing Sanger sequencing data. Phylogeographic results will be reported at a later date combined with microsatellite and mtDNA data (unpublished data).

### Ethics Statement

All necessary permits were obtained for the described field studies. The population collected in New South Wales was collected with our collaborator Maria Byrne under the authority of her Scientific Collecting Permit (P00/0015-5.0) with the New South Wales Government (Industry and Investment segment). With the exception above, no specific permits were required for the described field studies. These were non-invasive, non-lethal tissue samples taken from non-regulated, non-protected species on open, public lands.

### Marker Development

The five EPIC loci used in this study were developed for these two non-model organisms from various existing genomic resources (See Table 1). Primers amplifying an intron in the Glucose-6-Phosphate Isomerase gene (GPI) were modified from a previous study [31]. Primers amplifying introns in the TaTa Box Protein (TBP) and ATP Synthase Subunit Alpha (ATPSa) genes were modified from the available primers [32]. An intron in the Elongation Factor 1 Subunit Alpha (EFA1a) gene was developed using the expressed sequence tags (EST) of that gene from two related species *Asterina pectinifera* (DB414458) and *Patiria miniata* (AY580177). Finally, an intron in an unknown gene (called TP here) was developed from an EST from the testis of *Patiria miniata* (EX452619.1). All markers were designed to be between 400–600 basepairs (bp) of amplified product.

**Table 1:** List of amplicon primers and development methods.

Locus	Forward Primer	Reverse Primer	Development
	***P. exigua***		
**ATPSa**	TTTGCACCAGTGACCTTTTG	GCCCTTTGAGCTACAGTTCG	1
**EFA1a**	AAAGGAAGCCGCTGAGGTGAGT	CGATGACGGTGCAGTAATACC	2
**GPI**	GCCAAGCACTTTGTTGCCTT	TCCCAGAAGGGAAACATGTTATCCTTGTCG	3
**TBP**	TGTCAAGCAGTGCAACATTTC	GCTCCCTGATCCGCATAATA	1
**TP**	TTGGCTTATCCAGCGTTTCT	ATTCTCGCCCACTCAGTGAC	4
	***M. calcar***		
**ATPSa**	CGTGTCTTTTGCGGGTAAAC	AATGATGTTGGCACTTTTCG	1
**EFA1a**	AGTTAACCCAGTCACCAAGAGTCAWA	GGACCAAGTAGAAGGATTGCCCTC	2
**GPI**	GTGGCCCTGTCAACCAACG	TCCCARAARGGAAACATGTTWTCC	3
**TBP**	TGTTTCAAGCAATACAAAAAGGTT	GCACATTTGGCAACAGAAAT	5
**TP**	TGGCTCAAGTGGCTTATGTTC	ATGCAGCCCACCCTGATTAC	4

Primer sequences used for 454 sequencing are found in Table S1. 1- Modified from universal primers in [32], 2-Developed from ESTs from *Asterina pectinifera* (DB414458) and *Patiria miniata* (AY580177), 3-Developed from alignments of dog, frog, fish, and urchin GPI. See [31]. 4- Developed from an EST from the testis of *Patiria miniata* (EX452619.1), 5- Modified from universal primers in [32].

### Traditional Sanger Sequencing

PCRs were performed in 25 µL final volumes using Biomix Red 2× PCR mix (Bioline), forward and reverse primers, and H_2_O. Thermal cycling protocols followed the general form of 95°C denaturation for 3 minutes, then 35–40 cycles of 95°C for 30 seconds, 55–60°C for 45 seconds, and 60 seconds at 72°C, followed by a final extension of 10 min at 72°C.

*Parvulastra exigua* was sequenced directly (in both forward and reverse directions) for the loci GPI, EFA1a, and TP. Overall polymorphism was low allowing allelic states to be inferred by eye parsimoniously from mixed chromatograms using alleles from homozygous individuals. Results were also tested and validated with Phase [33]. Loci TBP and ATPSa were developed later and only used for 454 sequencing.

*Meridiastra calcar*, with extremely high levels of heterozygosity and polymorphism, required several different techniques to sequence and genotype three nDNA loci. Locus GPI was sequenced directly (forward and reverse directions). Most heterozygous individuals were cloned using the pJet Blunt End Cloning kit (Fermentas) with 4–8 clones sequenced per individual. Allelic states for a small number of individuals were inferred parsimoniously from mixed chromatograms using existing alleles from homozygous individuals and cloned alleles [31]. Loci EFA1a and TP were genotyped using allele-specific primers. Multiple and inconsistent banding patterns in gels of amplified EFA1a product along with inconsistent sequence quality from allele specific primers resulted in exclusion from the final analyses.

### 454 Project Design

**Figure 1:**
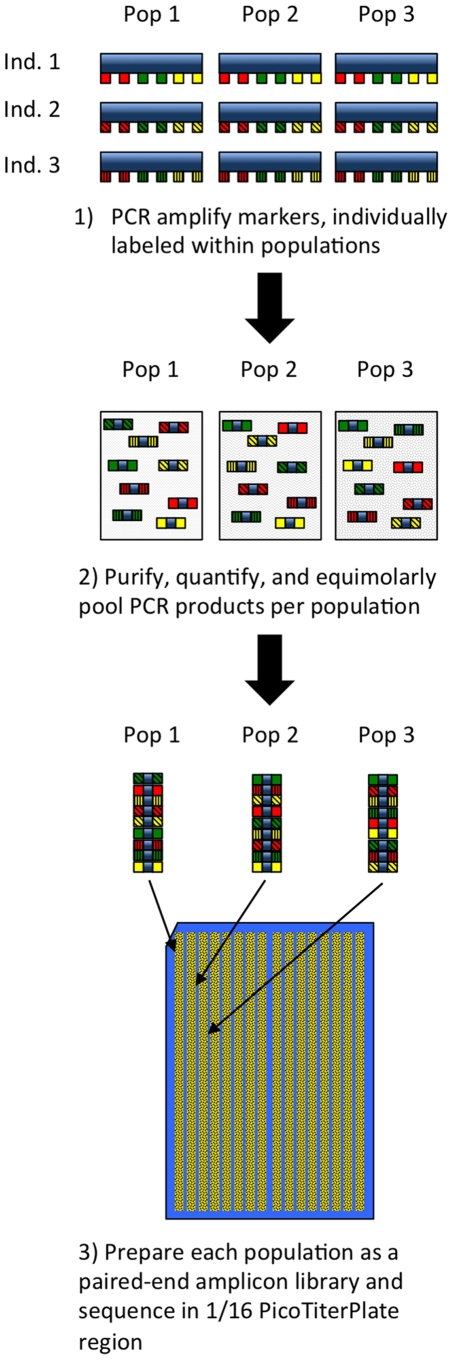
Graphical representation of the overall 454 experimental design and protocol.

The emulsion PCR [29]step of 454 sequencing amplifies a mixture of samples within one reaction. Therefore, samples require some sort of tagging within the product (serial tagging) or they need to be physically separated by a gasket on the 454 sequencing plate. We chose a combination of serial tagging and plate separation for multiplexing our samples, by dividing the plate into 16 regions (1 region per population) and tagging each individual within each population (Figure 1). As opposed to the serial tagging all samples, this design was chosen to minimize two factors 1) the cost of primer synthesis and 2) the impact of errant quantification and pooling of a single sample on overall library quality.

### 454 Primer Design

To allow for direct preparation of 454 sequencing libraries, the 454 Fusion Primers were added to the 5′ end of all template specific primers in accordance to Roche's primer design guidelines. To serialize our primers, we incorporated the Roche MID sequences between the Fusion primer sequence and our template specific primer for a final format of FusionPrimer-MID-EPICPrimer read in the 5′ to 3′ direction. We incorporated Fusion primer A in all forward primers and Fusion primer B in all reverse primers. Overall, 440 primers were synthesized (Table S1).

### 454 Library Preparation

Initially, all DNA extractions were aliquoted to 96 well PCR plates separated by population and species to ensure minimal chance of contamination and streamlined processing with multichannel pipettors. Adding the Fusion Primer and MID tail to the template specific primers, in addition to changing to a high fidelity, proofreading enzyme, significantly changed PCR reaction chemistry such that PCR reaction recipes and protocols required re-optimization to avoid primer dimers. PCRs were performed in 25 µL final volumes using AccuSure 2× PCR mix (Bioline), forward and reverse primers, H_2_O, and between 2.5 and 3.0 mM MgCl_2_ (this concentration of MgCl_2_, higher than the standard mix, was critical for successful amplification). Thermal cycling protocols followed the general form of 95°C denaturation for 10 minutes, then 35–40 cycles of 95°C for 30 seconds, 55–60°C for 45 seconds, and 1.5 minutes at 68°C, followed by a final extension of 20 min at 68°C. All reactions were checked by UV imaging of agarose gels for successful amplification. Products were then size selectively purified using AMPure beads (Agentcourt) using the manufacturer's protocol with the exception of a 0.8× bead to PCR reaction template ratio to ensure purification of all products below 300 bp. Amplified products were then quantified using a PicoGreen (Invitrogen) fluorescence assay on a SpectraMax M2 plate reader, using the standard SpectraMax protocol for a 96 well assay (Application Note #22). From these quantifications, the exact volume of each reaction to achieve 0.5 ng of amplified PCR product was calculated. This amount of each PCR product was then pooled by population (pooling both species together) to achieve 16 equimollarly-pooled libraries. After quality control runs on a BioAnalyzer 2100 (Agilent) performed by the sequencing facility and the sequencing of a small test library, we subsequently purified each library one additional time using the AMPure beads and protocol, this time with a 0.7 bead to DNA ratio.

### 454 Data Sorting and Assembly

Library files were imported into Geneious 5.4 [34]. All reads below 150 bp were removed and then libraries were sorted by MID tag and trimmed by quality score according to the default parameters of the program. Within each MID tag, contigs were assembled and then identified by locus specific primer sequences. Reads were discarded if: 1) the average quality score was below 30, 2) the whole primer sequence was not present in the read, or 3) the MID tag did not match at both ends of the product.

Random errors were common in many contigs and we called any bp that was below 75% consensus in an individual contig (1 Locus, 1 individual) heterozygous for that individual. However, if the variation was an INDEL polymorphism, especially a homopolymer (an extensive sequence of the same base pair), we used three criteria involving both majority consensus and the average quality score for the different lengths of polymer to make the call, and if there was less than 10× coverage for a locus, quality score was used exclusively. Ultimately, after all individual allelic states were determined, for a particular locus, they were aligned within populations for a final round of error checking. In this final alignment all singleton alleles (not 1× reads but alleles that were found only once in a population) were re-examined and, we assumed that it was far more likely if two alleles differed only by a single homopolymer INDEL that this was by sequencing artifact and subsequently edited the singleton to match.

If heterozygous base pairs were determined in the contig sequence, the contig was then sorted by that base pair. The two most common haplotypes were then selected as the two allelic states for that locus. Generally, these two alleles were obvious to the naked eye. A small number of reads associated with heterozygous loci appeared to be recombinants between the two dominant allele states. We assumed these to be PCR, emPCR, or sequencing artifacts and removed them from contigs.

### Alignment and Analysis

All sequences were aligned in Geneious using the MUSCLE algorithm with default parameters. Phylogenetic networks were constructed using the median joining and mp algorithms of NETWORK [35], [36] with default parameters to compare loci sequenced by both methods. All summary statistics were calculated in JMP 9.0 (SAS).

## Results

### 454 Library Quality and Coverage

**Figure 2:**
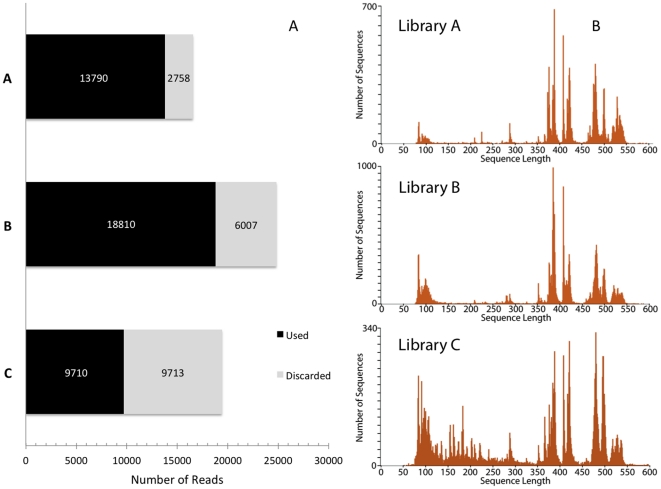
Visual Representation of Library Quality. A) Graph of the number of total reads by library, shaded by whether the reads were used or discarded. B) Histogram of reads by length for each library. Library A = New South Wales, Library B = Tasmania, and Library C = Southern Australia.

The three libraries generated over 60,788 reads with the average read over 350 bp in length (Figure 2). Of these reads, 42,310 were subsequently used for analysis. The number of reads and the percentage of reads discarded varied between libraries but increased numbers of shorter reads decreased the number of usable reads in the library (Figure 2). A large number of shorter reads present in Library C seriously impacted library quality, and more reads were discarded than actually used in analysis.

**Figure 3:**
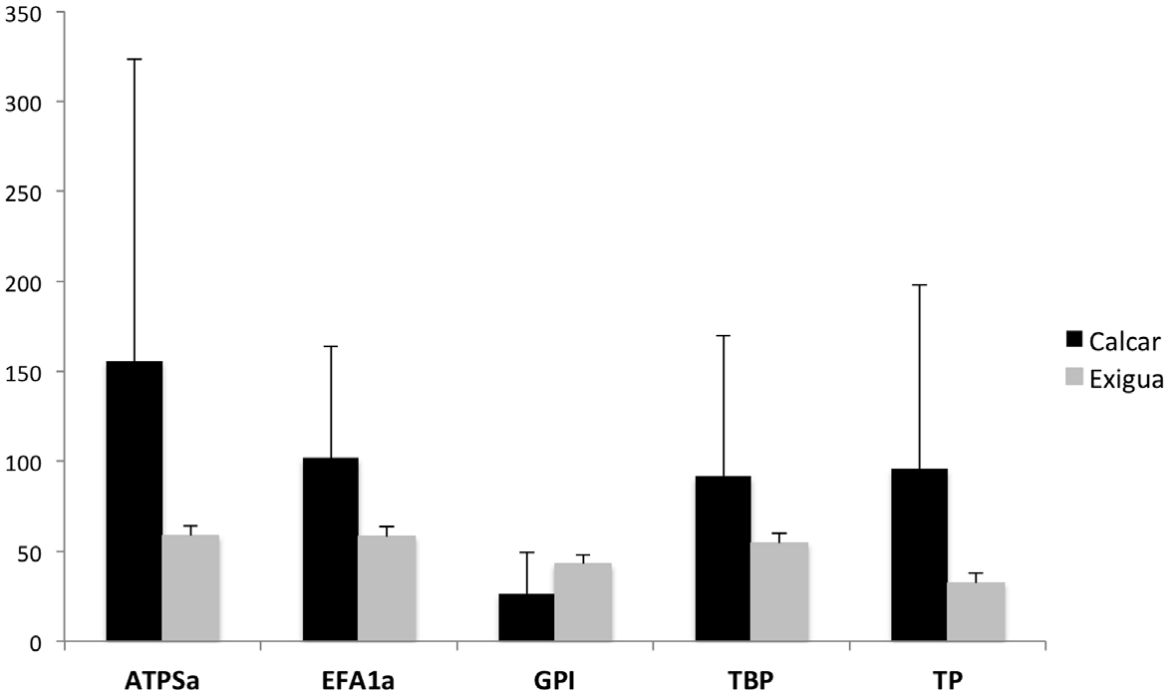
The average coverage per locus separated by species. *M. calcar* in black and *P. exigua* in gray. Average coverage was significantly different between species (Two sided t-test, t = −6.96455, p<0.0001) and between loci within species (*M. calcar* oneway ANOVA, F = 12.6416, p<0.0001; *P. exigua* oneway ANOVA, F = 4.2989, p<0.0021). Bars represent standard errors.

Despite the large number of discarded reads, the libraries achieved high levels of overall coverage. The average coverage per locus was 70.7× with over 97.2% of the loci sequenced with at least 1× coverage and over 95.2% sequenced to at least 5× coverage. Average coverage levels varied significantly by library (Oneway ANOVA F = 14.2435, p<0.0001) and appeared linked, as expected, to the number of reads. There were also significant differences in the coverage level between both species and loci (Figure 3). However, there was no significant difference in average coverage between different MID tags in either species (Oneway ANOVA, *M. calcar* F = 1.2893, p>0.1893; *P. exigua* F = 1.3451, p>14.87).

### Genetic Diversity and Sequencing Sensitivity

There is a large dichotomy in the genetic diversity of the two sampled species. *M. calcar* has both high levels of haplotype diversity and heterozygosity (Tables 2 and 3). On the other hand, *P. exigua* has considerably lower levels of haplotype diversity and little to no heterozygosity (Tables 2 and 3). In both species, 454 sequencing was more sensitive to detecting haplotypes and heterozygotes than Sanger sequencing methods, especially in markers with high diversity (Table 2).

**Table 2:** Genetic diversity and heterozygosity detected by both sequencing methods.

Species	Locus	Sanger Unique Alleles	454 Unique Alleles	Sanger Heterozygotes	454 Heterozygotes
*M. calcar*	GPI	45	74	11	22
*M. calcar*	TP	3	51	18	47
*P. exigua*	EFA1a	5	6	4	7
*P. exigua*	GPI	4	4	4	6
*P. exigua*	TP	3	4	7	8

Locus TP in *M. calcar* was only genotyped with sequence specific primers.

**Table 3:** Genetic diversity and heterozygosity of loci sequence only with the 454 method.

Species	Locus	454 Unique Alleles	454 Heterozygotes
*M. calcar*	ATPSA	59	26
*M. calcar*	EFA1a	63	31
*M. calcar*	TBP	47	28
*P. exigua*	ATPSA	3	0
*P. exigua*	TBP	1	0

*M. calcar* proved to be extremely genetically diverse at all sequenced loci. 454 sequencing was more sensitive than the method of “allele specific” genotyping used to genotype the locus TP. 454 sequencing detected an order of magnitude more unique haplotypes as well as nearly three times as many heterozygotes as “allele specific” genotyping (Table 2). The locus GPI proved to be difficult to sequence with both methods because of a second genomic priming site that was present in about 40% of individuals. Despite several rounds of primer design, PCR optimization, and cloning, this mispriming could not be removed and sometimes prevented the sequencing of the actual GPI locus. 454 sequencing proved more capable of handling this type of technical difficulty. Overall, 17 specimens failed to sequence the GPI locus; however, only six samples failed using 454 sequencing and five of these failed with both methods. At the allelic level, Sanger sequencing failed to detect 29 alleles whereas 454 sequencing failed to detect only one allele that was present in Sanger sequences. Overall, 454 sequencing proved to be nearly twice as sensitive as Sanger sequencing in detecting both genotypic and haplotypic variation.

The lower genetic diversity of *P. exigua* allowed for the direct sequencing of three loci using Sanger sequencing, and across all individuals and loci (N = 291), only seven differed between the two sequencing methods. In six of the seven differences, the direct Sanger sequencing approach failed to detect alleles that were revealed in the 454 approach, so that heterozygotes were underrepresented with the former method (Table 4). For these six specimens, coverage is relatively high with near equal proportions of both alleles in the overall coverage, making it unlikely that any represent contamination or PCR errors (Table 4). In the seventh individual, 454 sequencing failed to detect an allele in a heterozygous individual recovered by Sanger sequencing. In this final case, however, there is 163× coverage by 454, and this indicates that the heterozygous result from Sanger sequencing may be erroneous. Even at low levels of DNA polymorphism and heterozygosity, 454 sequencing appears to be more sensitive than the traditional approach of direct Sanger sequencing confirmed by cloning.

**Table 4:** Loci for which 454 sequencing detected additional variation in *Parvulastra exigua*.

Sample	Locus	Total Coverage	Allele 1 Coverage	Allele 2 Coverage	Allele 1 Proportion of Total Coverage
B01	EFA1a	76	44	32	0.578947368
B10	EFA1a	52	31	21	0.596153846
B18	EFA1a	92	56	36	0.608695652
A109	GPI	50	32	18	0.507936508
A117	TP	34	22	12	0.628571429
B05	TP	37	27	10	0.72972973

Despite differences in sensitivity, haplotypes generated by both sequencing methods were virtually identical. In *M. calcar* (where only locus GPI was cloned), haplotypes were on average 99.56% identical at the base pair level. In *P. exigua*, haplotypes across all three loci (GPI, EFA1a, and TP) were on average 99.89% identical at the base pair level, with the majority of differences lying in previously undetected alleles. In other words, all alleles detect by both methods of sequencing were 100% identical, with only two previously undetected alleles discovered by 454 sequencing. Recombinant reads were a common feature of many heterozygous loci; however, these reads often occur at small percentages of the total coverage, averaging only 12.5% across all heterozygous loci. They were easily identified and removed by eye, as described in the Methods section.

### Phylogeographic Results

**Figure 4:**
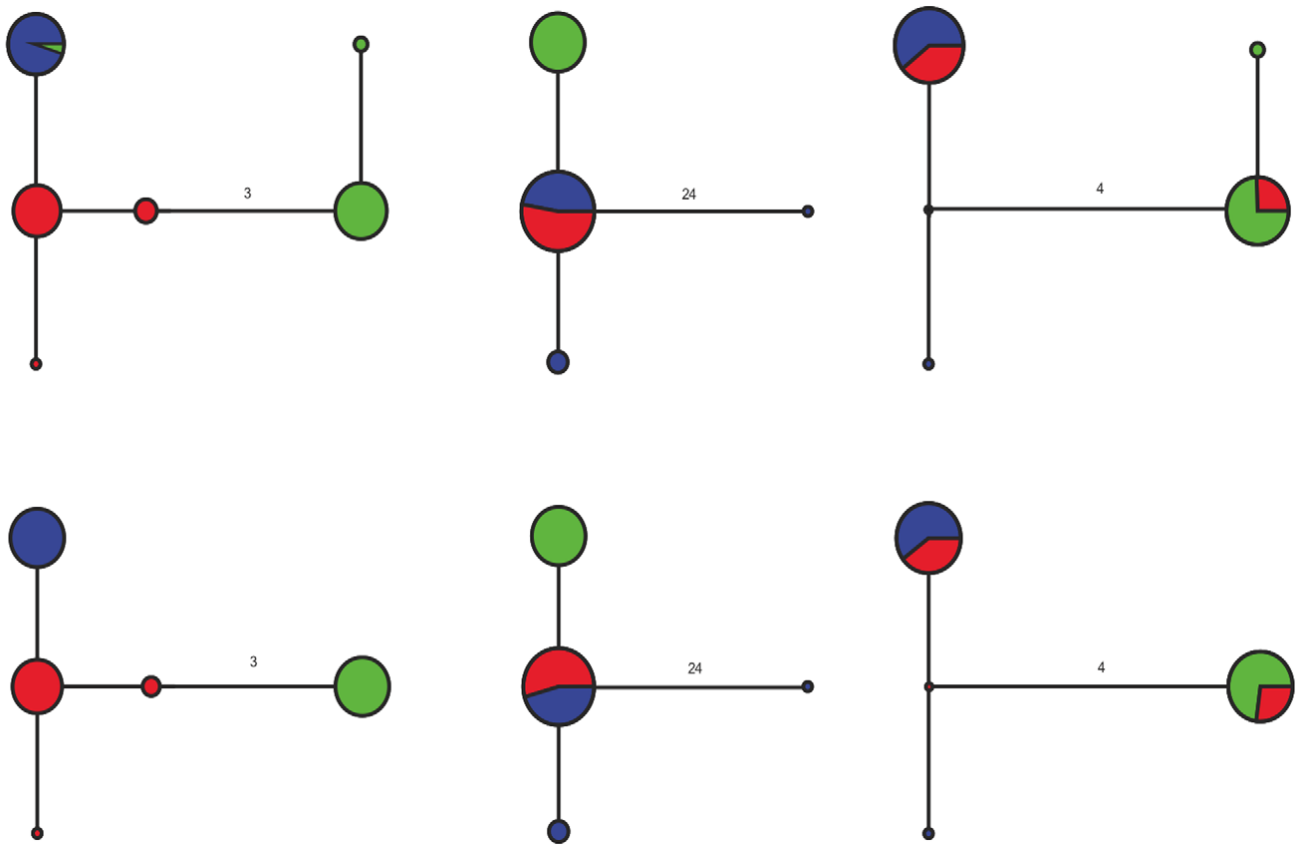
Allelic networks for nDNA loci in *Parvulastra exigua*. Networks on top were obtained via 454 sequencing and those on bottom from direct Sanger Sequencing. Loci from left to right are EFA1a, GPI, and TP. Samples from New South Wales are colored in blue, Tasmania in red, and Southern Australia in green. Numbers on network branches represent the number of mutational steps (including INDEL) between alleles.

High levels of allelic and nucleotide diversity in the locus GPI of *M. calcar* made networks for both methods largely uninformative, although rather similar in outward appearance (Figure S1). However, networks from the three loci sequenced by both methods (GPI, EFA1a, and TP) in *P. exigua* showed that both data sets would lead to largely similar phylogeographic conclusions (Figure 4). 454 sequencing did detect additional alleles in two of the three loci, and also detected that New South Whales and Southern Australia share allele that was not detected by Sanger sequencing.

### Time and Cost

It took four researchers approximately six months of dedicated lab time each to complete the initial effort of sequencing and genotyping six different nDNA loci (three per species). In the end however, only four of these six loci were sequenced completely due to complications. By comparison, all lab work, including primer development and optimization, for the entire 454 sequencing phase of the project was completed by one researcher in approximately six months of lab time (Table 5). There was some overlap of primer development between project phases and these time differences do not take into account the differences in sequence processing time. Regardless, our best estimate is that 454 sequencing as outlined here is three to four times more time efficient (in terms of cost and manpower) than traditional Sanger-based methods for sequencing multiple nDNA markers.

**Table 5:** Approximate lab time needed to complete each sequencing objective.

Method	Number of Loci	Number of Researchers	Lab Time	Total Labor Time
454	10	1	6 months	6 months
Sanger	6 (4)	4	6 months	24 months

Total Labor Time was calculated as the amount of time it would take one researcher to complete the research objective.

The major costs of the targeted 454 method is the full plate of sequencing which includes library quality testing, quantification, and emPCR, and the cost for 400 primers (2 labeled primers each for 20 individuals across 2 species and 5 loci). This puts the total cost at $24,560 to sequence approximately 16 populations or 3200 individual nDNA loci. At $4.00 per individual sequence, the cost to Sanger sequence 3200 loci in the forward and reverse direction is approximately $25,725, already above the 454 price point. Additionally, if any cloning becomes necessary this cost savings quickly increases (Table 6). Overall, in species with moderate to high levels of genetic diversity and heterozygosity, 454 sequencing will be a much more cost effective way to sequence multiple nDNA intron loci.

**Table 6:** Costs of sequencing 3200 loci.

	454	F+R sequencing	F+R, 12.5% Cloned 5X	F+R, 25% Cloned 5X	F+R, 50% Cloned 5X
Total Cost for 400 primers	$12,137	$125	$125	$125	$125
Total Cost for Full plate sequencing	$12,423	$25,600	$38,600	$51,600	$77,600
Total Major Costs	$24,560	$25,725	$38,725	$51,725	$77,725

Sequencing costs were estimated at $4.00 per Sanger read and five reads per cloned locus. Additionally, a $12.50 cost of cloning (based on $245 for 20 reactions of the Promega pGEM-T Easy Vector System) was added per sample. 12.5%, 25% and 50% are in reference to the proportion of individual loci that would need to be cloned to discern individual alleles.

## Discussion

### Relative Performance of Markers and Species

Based on this experimental design, all markers and species should, theoretically, have very similar relative levels of coverage. However, there were significant differences in coverage between the two different species and between markers within species even though all amplicons were quantified and pooled equimollarly within libraries. The difference between species is perhaps due to the level of genetic diversity. Primer design for the diverse *M. calcar* was difficult leading to less specific primers required to ensure broad amplification. Specifically, two loci for *M. calcar* had degenerate primers pairs (EFA1a and GPI), and these were also the loci that had high frequency nonspecific priming. It is possible that these non-target products helped to increase the relative amounts of *M. calcar* DNA in the post emPCR products.

The differences between loci within species appeared to be of separate causes. In the less variable *P. exigua*, coverage depended on the size of the amplicon, with the two largest loci (GPI and TP) receiving less coverage than the other three which had similar levels of coverage. These differences between loci were even more pronounced in the more variable *M. calcar*. Locus GPI had depressed levels of coverage, most likely due to the high levels of non-specific binding of the 6× degenerate primer pair. It is unclear as to why locus ATPS would have relatively high levels of coverage.

### Is 454 sequencing more sensitive to genetic variation or does it artificially inflate it?

In almost every comparison, 454 sequencing detected more allelic variants and more heterozygous loci than traditional Sanger sequencing. We interpret this as 454 sequencing being more sensitive to genetic polymorphism; however, it could be argued that 454 sequencing is less accurate per nucleotide and is inflating estimates of genetic variation. Our simple allelic network comparison suggests that phylogeographic interpretations would not drastically change either way, but several lines of evidence suggest that 454 sequencing is not artificially enhancing genetic variation.

The first is the high level of shared identity (over 99% in both species) in alleles generated by both methods. Sequencing error rates for the GS-FLX Titanium system have been estimated at ∼1%; however, even if the error rate was an order of magnitude higher (∼10%) we would still expect to find, with over 99% confidence, a majority of correct sequences in a sample of five reads [37]. For heterozygous loci, with two correct sequences and an equal ratio within the sample, we could expect to have this same accuracy for both alleles in a sample of 10. On average, heterozygous loci in our libraries had 107× coverage with an average allelic ratio of 13∶9, giving us a very high likelihood of recovering the two true alleles by simple majority consensus.

*P. exigua*, with low levels of nucleotide diversity and heterozygosity, presents an ideal test for artificial inflation of genetic diversity by 454 sequencing. Only seven individual loci differed between sequencing methods (Table 4), and most importantly, alleles detected by both methods were identical. The main difference between the methods was the detection of a second allele, or in other words, a heterozygous individual versus a homozygous one. In only two of these six cases, the previously undetected allele was novel. All of these loci had allelic ratios close to 1∶1, meaning that the second previously undetected allele was unlikely to be a small amount of contamination from another sample. PCR errors are also unlikely because of the proof-reading DNA polymerase used for all 454 reactions. Moreover, one locus, TBP, was completely fixed for one allele across all samples, again providing evidence that 454 sequencing is not artificially inflating genetic diversity.

Lastly, while recombinant reads were detected in several heterozygous samples, they were on average a low percentage of reads (12.5%) within each locus. We believe that these recombinant reads are generated during the initial PCR amplification because of heterozygous chimeric sequences [38]. In other words, this is not inherent to 454 sequencing, and would only effect heterozygous loci. It would not be responsible for adding any allelic variation to homozygous loci, only adding a small percentage of erroneous reads to an already heterozygous locus. In *P. exigua*, no additional allelic variation was detected in GPI (the directly sequenced loci) within homozygotes or heterozygotes, and in *M. calcar* the majority of new allelic variation, 72.4%, was detected in samples that were previously believed to be homozygous. Although there were some alleles that differed between methods within heterozygous loci, it is worth considering that these loci in Sanger sequencing were sequenced at 4–6× clonal coverage, almost two orders of magnitude lower coverage than the average 454 locus. Additionally, PCR amplifications for Sanger sequencing used a non-proofreading DNA polymerase and would be more susceptible to PCR errors. In short, available data provides no evidence that recombination is inflating genetic diversity detected by 454 sequencing. Considering the high levels shared identity in alleles, the low incidence of new alleles detected in *P. exigua*, and the low levels of recombinant reads compared to overall coverage, our results indicate that targeted 454 sequencing represents a more sensitive approach to sequencing and detecting genetic polymorphisms in nDNA loci.

### Possible Modifications and Extensions

Though several quality control steps were taken in this protocol, Library C still had a large number of short, low quality reads. A small number of samples that were not completely purified and subsequently included could have been responsible for these reads. Although we performed some random spot checks, we would now advise a check of PCR reactions with gel electrophoresis after the SPRI purification to ensure library quality. It may also be worth considering a second quantification step immediately after the first. While the SpectraMax protocol involves the averaging of 10 different reads, pipetting errors in PicoGreen preparations are inevitable. Considering how crucial equimolar pooling is to the procedure, and how little time it takes to perform the quantifications, it is an easy additional quality control step.

We achieved at least 5× coverage for over 95% of the loci surveyed, and therefore we believe that this design maximizes the number of nDNA loci that can be simultaneously sequenced in one 454 sequencing run. Improvements may be possible to the purification and quantification protocols, as discussed above, but based on the published read numbers, these changes are expected to generate less than 10% improvement over our results. This improvement may allow for the inclusion of more individuals per population, but is unlikely to allow for the inclusion of more loci.

One possible way to improve this protocol would be to perform serial sample tagging in-house instead of ordering primers with tags incorporated. Other published protocols could easily be modified to accomplish this [39], [40]. However, as stated in Meyer et al. [39], these procedures would also necessitate a qPCR step to ensure quantification of amplicons that were successfully tagged and contain the full FusionPrimer-SerialTag-Amplicon-SpecificPrimer. Another way to make this procedure more cost efficient would be to design locus amplicons that are short enough to incorporate both forward and reverse primers in each read. If this is done, then combinations of different tags on the forward and reverse primers can be used to identify individuals instead of the same tag on both primers. Lastly, we chose to use the PAGE purification (Invitrogen) process to purify our primers to safeguard the purity and length of each primer; however, significant cost savings could be achieved with a less expensive purification method. The high coverage of most loci in this study suggests that errors in the primer synthesis could be tolerated, especially with long serial tags. Each of these suggested modifications would save dramatically on primer costs and make the price point of targeted 454 nDNA sequencing even lower.

### Comparisons to other next-generation sequencing methods

The vast array of next-generation sequencing technology has a number of different applications to population and conservation genetics [28], [41]. However, few of these technologies have been applied in phylogeographic studies, with the exception of Emerson et al. [42] that used restriction-site-associated DNA tags (RAD tags) [43], [44] to determine the evolutionary relationship between recently diverged populations of pitcher plant mosquitos. This methodology can genotype multiple populations at thousands of SNP loci simultaneously, but have limited ability to survey large sample sizes within populations because of the cost. For example, Emerson et al. genotyped 21 different population at ∼13,627 different SNPs but with only 6 individuals per population the authors were forced to restrict their analysis to 3,741 SNPs “that were fixed within at least two populations and were variable among populations” and generate one consensus sequence per locus per population [42]. Our targeted 454 sequencing methodology offers a compromise with the ability to sequence a reasonable sample size (20 individuals) from one population for 10 different nDNA loci (∼400 bp each) in 1/16^th^ of a plate of 454 sequencing, at lower cost than a RAD tag analysis. In other words, 1/16^th^ plate of 454 sequencing is less expensive than a single lane of Illumina sequencing at current market prices, and only ∼16 individuals can be RAD tagged in one lane [44], [45]. We believe the approach outlined here generates equally high quality genetic data with larger sample sizes and is a good compromise between cost and depth of genomic sampling for phylogeographic analyses.

### Conclusions

The results from this study indicate that targeted 454 sequencing is an accurate and sensitive approach to nDNA intron sequencing that is more time and cost effective than traditional Sanger sequencing based methods. Using 454 sequencing technology, we were able to simultaneously sequence 5 nDNA loci in each of two different non-model organisms across 16 populations at relatively high levels of coverage. High coverage of loci greatly simplified the task of determining allelic state, even in loci with small levels of non-target amplification. Additionally, we showed that phylogeographic interpretations of the two different would be largely similar. In conclusion, targeted 454 sequencing provides the means to incorporate large nDNA data sets into phylogeographic studies, even with highly polymorphic, non-model organisms with low levels of non-target amplification in a highly polymorphic species.

## Supporting Information

Figure S1 **Allelic networks for GPI in** ***Meridiastra calcar*** **.** Network on top was obtained via 454 sequencing and the bottom from direct Sanger Sequencing. Samples from New South Wales are colored in blue, Tasmania in red, and Southern Australia in green. Numbers on network branches represent the number of mutational steps (including INDEL) between alleles.
(DOCX)

Table S1 **Complete List of Primers.**
(DOCX)
