# Supplementary figures and images for: Next-Generation Phylogeography: A Targeted Approach for Multilocus Sequencing of Non-Model Organisms
Source: PLoS One. 2012 Mar 28;7(3):e34241. doi: 10.1371/journal.pone.0034241 (PMC3314618; doi:10.1371/journal.pone.0034241)

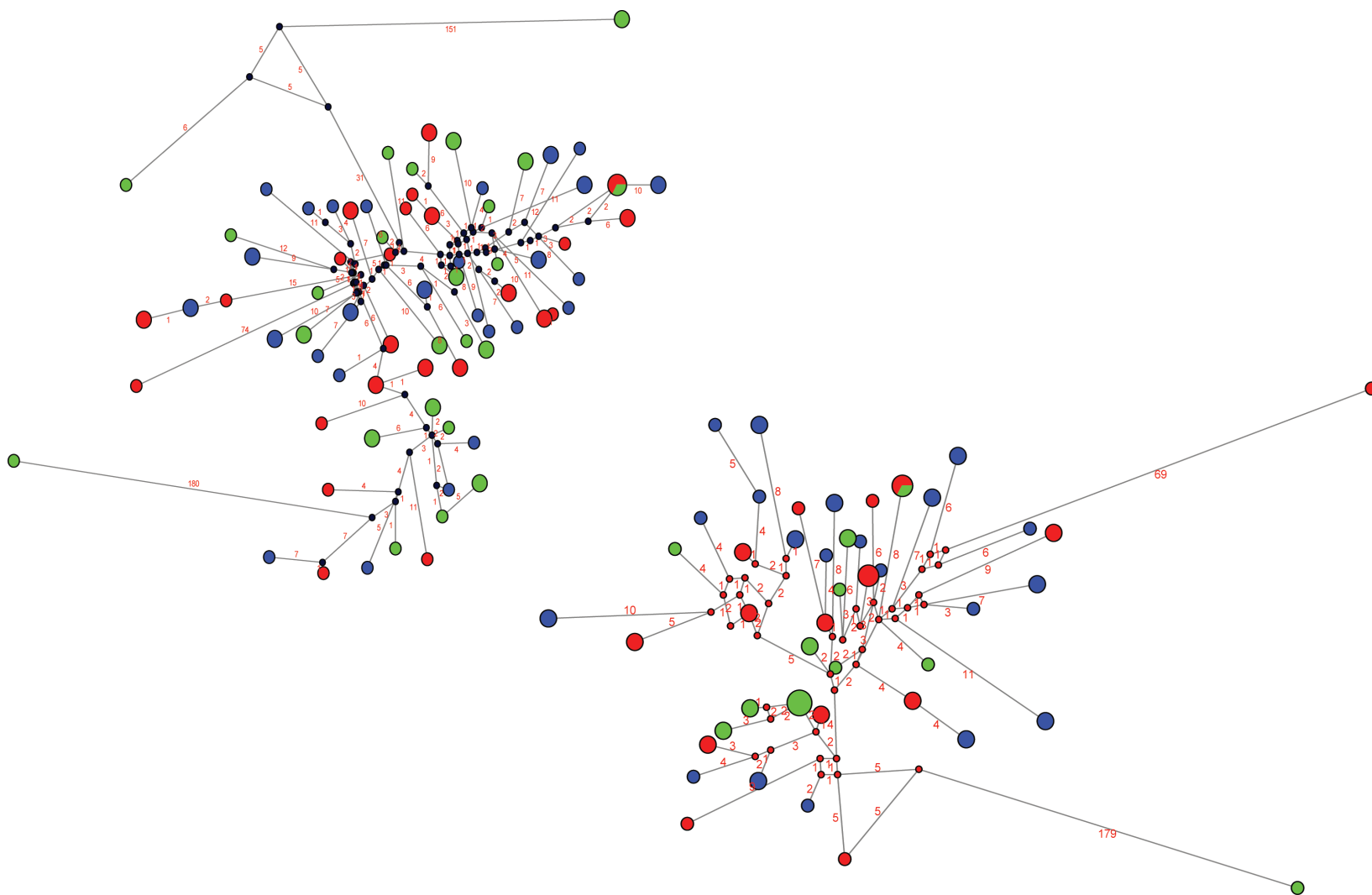

Supplement: Figure S1 — Allelic networks for GPI in Meridiastra calcar . Network on top was obtained via 454 sequencing and the bottom from direct Sanger Sequencing. Samples from New South Wales are colored in blue, Tasmania in red, and Southern Australia in green. Numbers on network branches represent the number of mutational steps (including INDEL) between alleles. (DOCX) [file pone.0034241.s001.docx]
