# Supplementary material for: Next-Generation Phylogeography: A Targeted Approach for Multilocus Sequencing of Non-Model Organisms
Source: PLoS One. 2012 Mar 28;7(3):e34241. doi: 10.1371/journal.pone.0034241 (PMC3314618; doi:10.1371/journal.pone.0034241)
Supplement: Table S1 — Complete List of Primers. (DOCX) [file pone.0034241.s002.docx]

| CALTPFUSAMID1 | CGTATCGCCTCCCTCGCGCCATCAGACGAGTGCGTTGGCTCAAGTGGCTTATGTTC |
| --- | --- |
| CALTPFUSAMID2 | CGTATCGCCTCCCTCGCGCCATCAGACGCTCGACATGGCTCAAGTGGCTTATGTTC |
| CALTPFUSAMID3 | CGTATCGCCTCCCTCGCGCCATCAGAGACGCACTCTGGCTCAAGTGGCTTATGTTC |
| CALTPFUSAMID4 | CGTATCGCCTCCCTCGCGCCATCAGAGCACTGTAGTGGCTCAAGTGGCTTATGTTC |
| CALTPFUSAMID5 | CGTATCGCCTCCCTCGCGCCATCAGATCAGACACGTGGCTCAAGTGGCTTATGTTC |
| CALTPFUSAMID6 | CGTATCGCCTCCCTCGCGCCATCAGATATCGCGAGTGGCTCAAGTGGCTTATGTTC |
| CALTPFUSAMID7 | CGTATCGCCTCCCTCGCGCCATCAGCGTGTCTCTATGGCTCAAGTGGCTTATGTTC |
| CALTPFUSAMID8 | CGTATCGCCTCCCTCGCGCCATCAGCTCGCGTGTCTGGCTCAAGTGGCTTATGTTC |
| CALTPFUSAMID10 | CGTATCGCCTCCCTCGCGCCATCAGTCTCTATGCGTGGCTCAAGTGGCTTATGTTC |
| CALTPFUSAMID11 | CGTATCGCCTCCCTCGCGCCATCAGTGATACGTCTTGGCTCAAGTGGCTTATGTTC |
| CALTPFUSAMID13 | CGTATCGCCTCCCTCGCGCCATCAGCATAGTAGTGTGGCTCAAGTGGCTTATGTTC |
| CALTPFUSAMID14 | CGTATCGCCTCCCTCGCGCCATCAGCGAGAGATACTGGCTCAAGTGGCTTATGTTC |
| CALTPFUSAMID15 | CGTATCGCCTCCCTCGCGCCATCAGATACGACGTATGGCTCAAGTGGCTTATGTTC |
| CALTPFUSAMID16 | CGTATCGCCTCCCTCGCGCCATCAGTCACGTACTATGGCTCAAGTGGCTTATGTTC |
| CALTPFUSAMID17 | CGTATCGCCTCCCTCGCGCCATCAGCGTCTAGTACTGGCTCAAGTGGCTTATGTTC |
| CALTPFUSAMID18 | CGTATCGCCTCCCTCGCGCCATCAGTCTACGTAGCTGGCTCAAGTGGCTTATGTTC |
| CALTPFUSAMID19 | CGTATCGCCTCCCTCGCGCCATCAGTGTACTACTCTGGCTCAAGTGGCTTATGTTC |
| CALTPFUSAMID20 | CGTATCGCCTCCCTCGCGCCATCAGACGACTACAGTGGCTCAAGTGGCTTATGTTC |
| CALTPFUSAMID21 | CGTATCGCCTCCCTCGCGCCATCAGCGTAGACTAGTGGCTCAAGTGGCTTATGTTC |
| CALTPFUSAMID22 | CGTATCGCCTCCCTCGCGCCATCAGTACGAGTATGTGGCTCAAGTGGCTTATGTTC |
| CALTPFUSAMID23 | CGTATCGCCTCCCTCGCGCCATCAGTACTCTCGTGTGGCTCAAGTGGCTTATGTTC |
| CALTPFUSAMID24 | CGTATCGCCTCCCTCGCGCCATCAGTAGAGACGAGTGGCTCAAGTGGCTTATGTTC |
| CALTPFUSBMID1 | CTATGCGCCTTGCCAGCCCGCTCAGACGAGTGCGTATGCAGCCCACCCTGATTAC |
| CALTPFUSBMID2 | CTATGCGCCTTGCCAGCCCGCTCAGACGCTCGACAATGCAGCCCACCCTGATTAC |
| CALTPFUSBMID3 | CTATGCGCCTTGCCAGCCCGCTCAGAGACGCACTCATGCAGCCCACCCTGATTAC |
| CALTPFUSBMID4 | CTATGCGCCTTGCCAGCCCGCTCAGAGCACTGTAGATGCAGCCCACCCTGATTAC |
| CALTPFUSBMID5 | CTATGCGCCTTGCCAGCCCGCTCAGATCAGACACGATGCAGCCCACCCTGATTAC |
| CALTPFUSBMID6 | CTATGCGCCTTGCCAGCCCGCTCAGATATCGCGAGATGCAGCCCACCCTGATTAC |
| CALTPFUSBMID7 | CTATGCGCCTTGCCAGCCCGCTCAGCGTGTCTCTAATGCAGCCCACCCTGATTAC |
| CALTPFUSBMID8 | CTATGCGCCTTGCCAGCCCGCTCAGCTCGCGTGTCATGCAGCCCACCCTGATTAC |
| CALTPFUSBMID10 | CTATGCGCCTTGCCAGCCCGCTCAGTCTCTATGCGATGCAGCCCACCCTGATTAC |
| CALTPFUSBMID11 | CTATGCGCCTTGCCAGCCCGCTCAGTGATACGTCTATGCAGCCCACCCTGATTAC |
| CALTPFUSBMID13 | CTATGCGCCTTGCCAGCCCGCTCAGCATAGTAGTGATGCAGCCCACCCTGATTAC |
| CALTPFUSBMID14 | CTATGCGCCTTGCCAGCCCGCTCAGCGAGAGATACATGCAGCCCACCCTGATTAC |
| CALTPFUSBMID15 | CTATGCGCCTTGCCAGCCCGCTCAGATACGACGTAATGCAGCCCACCCTGATTAC |
| CALTPFUSBMID16 | CTATGCGCCTTGCCAGCCCGCTCAGTCACGTACTAATGCAGCCCACCCTGATTAC |
| CALTPFUSBMID17 | CTATGCGCCTTGCCAGCCCGCTCAGCGTCTAGTACATGCAGCCCACCCTGATTAC |
| CALTPFUSBMID18 | CTATGCGCCTTGCCAGCCCGCTCAGTCTACGTAGCATGCAGCCCACCCTGATTAC |
| CALTPFUSBMID19 | CTATGCGCCTTGCCAGCCCGCTCAGTGTACTACTCATGCAGCCCACCCTGATTAC |
| CALTPFUSBMID20 | CTATGCGCCTTGCCAGCCCGCTCAGACGACTACAGATGCAGCCCACCCTGATTAC |
| CALTPFUSBMID21 | CTATGCGCCTTGCCAGCCCGCTCAGCGTAGACTAGATGCAGCCCACCCTGATTAC |
| CALTPFUSBMID22 | CTATGCGCCTTGCCAGCCCGCTCAGTACGAGTATGATGCAGCCCACCCTGATTAC |
| CALTPFUSBMID23 | CTATGCGCCTTGCCAGCCCGCTCAGTACTCTCGTGATGCAGCCCACCCTGATTAC |
| CALTPFUSBMID24 | CTATGCGCCTTGCCAGCCCGCTCAGTAGAGACGAGATGCAGCCCACCCTGATTAC |
| EXITPFUSAMID1 | CGTATCGCCTCCCTCGCGCCATCAGACGAGTGCGTTTGGCTTATCCAGCGTTTCT |
| EXITPFUSAMID2 | CGTATCGCCTCCCTCGCGCCATCAGACGCTCGACATTGGCTTATCCAGCGTTTCT |
| EXITPFUSAMID3 | CGTATCGCCTCCCTCGCGCCATCAGAGACGCACTCTTGGCTTATCCAGCGTTTCT |
| EXITPFUSAMID4 | CGTATCGCCTCCCTCGCGCCATCAGAGCACTGTAGTTGGCTTATCCAGCGTTTCT |
| EXITPFUSAMID5 | CGTATCGCCTCCCTCGCGCCATCAGATCAGACACGTTGGCTTATCCAGCGTTTCT |
| EXITPFUSAMID6 | CGTATCGCCTCCCTCGCGCCATCAGATATCGCGAGTTGGCTTATCCAGCGTTTCT |
| EXITPFUSAMID7 | CGTATCGCCTCCCTCGCGCCATCAGCGTGTCTCTATTGGCTTATCCAGCGTTTCT |
| EXITPFUSAMID8 | CGTATCGCCTCCCTCGCGCCATCAGCTCGCGTGTCTTGGCTTATCCAGCGTTTCT |
| EXITPFUSAMID10 | CGTATCGCCTCCCTCGCGCCATCAGTCTCTATGCGTTGGCTTATCCAGCGTTTCT |
| EXITPFUSAMID11 | CGTATCGCCTCCCTCGCGCCATCAGTGATACGTCTTTGGCTTATCCAGCGTTTCT |
| EXITPFUSAMID13 | CGTATCGCCTCCCTCGCGCCATCAGCATAGTAGTGTTGGCTTATCCAGCGTTTCT |
| EXITPFUSAMID14 | CGTATCGCCTCCCTCGCGCCATCAGCGAGAGATACTTGGCTTATCCAGCGTTTCT |
| EXITPFUSAMID15 | CGTATCGCCTCCCTCGCGCCATCAGATACGACGTATTGGCTTATCCAGCGTTTCT |
| EXITPFUSAMID16 | CGTATCGCCTCCCTCGCGCCATCAGTCACGTACTATTGGCTTATCCAGCGTTTCT |
| EXITPFUSAMID17 | CGTATCGCCTCCCTCGCGCCATCAGCGTCTAGTACTTGGCTTATCCAGCGTTTCT |
| EXITPFUSAMID18 | CGTATCGCCTCCCTCGCGCCATCAGTCTACGTAGCTTGGCTTATCCAGCGTTTCT |
| EXITPFUSAMID19 | CGTATCGCCTCCCTCGCGCCATCAGTGTACTACTCTTGGCTTATCCAGCGTTTCT |
| EXITPFUSAMID20 | CGTATCGCCTCCCTCGCGCCATCAGACGACTACAGTTGGCTTATCCAGCGTTTCT |
| EXITPFUSAMID21 | CGTATCGCCTCCCTCGCGCCATCAGCGTAGACTAGTTGGCTTATCCAGCGTTTCT |
| EXITPFUSAMID22 | CGTATCGCCTCCCTCGCGCCATCAGTACGAGTATGTTGGCTTATCCAGCGTTTCT |
| EXITPFUSAMID23 | CGTATCGCCTCCCTCGCGCCATCAGTACTCTCGTGTTGGCTTATCCAGCGTTTCT |
| EXITPFUSAMID24 | CGTATCGCCTCCCTCGCGCCATCAGTAGAGACGAGTTGGCTTATCCAGCGTTTCT |
| EXITPFUSBMID1 | CTATGCGCCTTGCCAGCCCGCTCAGACGAGTGCGTATTCTCGCCCACTCAGTGAC |
| EXITPFUSBMID2 | CTATGCGCCTTGCCAGCCCGCTCAGACGCTCGACAATTCTCGCCCACTCAGTGAC |
| EXITPFUSBMID3 | CTATGCGCCTTGCCAGCCCGCTCAGAGACGCACTCATTCTCGCCCACTCAGTGAC |
| EXITPFUSBMID4 | CTATGCGCCTTGCCAGCCCGCTCAGAGCACTGTAGATTCTCGCCCACTCAGTGAC |
| EXITPFUSBMID5 | CTATGCGCCTTGCCAGCCCGCTCAGATCAGACACGATTCTCGCCCACTCAGTGAC |
| EXITPFUSBMID6 | CTATGCGCCTTGCCAGCCCGCTCAGATATCGCGAGATTCTCGCCCACTCAGTGAC |
| EXITPFUSBMID7 | CTATGCGCCTTGCCAGCCCGCTCAGCGTGTCTCTAATTCTCGCCCACTCAGTGAC |
| EXITPFUSBMID8 | CTATGCGCCTTGCCAGCCCGCTCAGCTCGCGTGTCATTCTCGCCCACTCAGTGAC |
| EXITPFUSBMID10 | CTATGCGCCTTGCCAGCCCGCTCAGTCTCTATGCGATTCTCGCCCACTCAGTGAC |
| EXITPFUSBMID11 | CTATGCGCCTTGCCAGCCCGCTCAGTGATACGTCTATTCTCGCCCACTCAGTGAC |
| EXITPFUSBMID13 | CTATGCGCCTTGCCAGCCCGCTCAGCATAGTAGTGATTCTCGCCCACTCAGTGAC |
| EXITPFUSBMID14 | CTATGCGCCTTGCCAGCCCGCTCAGCGAGAGATACATTCTCGCCCACTCAGTGAC |
| EXITPFUSBMID15 | CTATGCGCCTTGCCAGCCCGCTCAGATACGACGTAATTCTCGCCCACTCAGTGAC |
| EXITPFUSBMID16 | CTATGCGCCTTGCCAGCCCGCTCAGTCACGTACTAATTCTCGCCCACTCAGTGAC |
| EXITPFUSBMID17 | CTATGCGCCTTGCCAGCCCGCTCAGCGTCTAGTACATTCTCGCCCACTCAGTGAC |
| EXITPFUSBMID18 | CTATGCGCCTTGCCAGCCCGCTCAGTCTACGTAGCATTCTCGCCCACTCAGTGAC |
| EXITPFUSBMID19 | CTATGCGCCTTGCCAGCCCGCTCAGTGTACTACTCATTCTCGCCCACTCAGTGAC |
| EXITPFUSBMID20 | CTATGCGCCTTGCCAGCCCGCTCAGACGACTACAGATTCTCGCCCACTCAGTGAC |
| EXITPFUSBMID21 | CTATGCGCCTTGCCAGCCCGCTCAGCGTAGACTAGATTCTCGCCCACTCAGTGAC |
| EXITPFUSBMID22 | CTATGCGCCTTGCCAGCCCGCTCAGTACGAGTATGATTCTCGCCCACTCAGTGAC |
| EXITPFUSBMID23 | CTATGCGCCTTGCCAGCCCGCTCAGTACTCTCGTGATTCTCGCCCACTCAGTGAC |
| EXITPFUSBMID24 | CTATGCGCCTTGCCAGCCCGCTCAGTAGAGACGAGATTCTCGCCCACTCAGTGAC |
| EXITBPFUSAMID1 | CGTATCGCCTCCCTCGCGCCATCAGACGAGTGCGTTGTCAAGCAGTGCAACATTTC |
| EXITBPFUSAMID2 | CGTATCGCCTCCCTCGCGCCATCAGACGCTCGACATGTCAAGCAGTGCAACATTTC |
| EXITBPFUSAMID3 | CGTATCGCCTCCCTCGCGCCATCAGAGACGCACTCTGTCAAGCAGTGCAACATTTC |
| EXITBPFUSAMID4 | CGTATCGCCTCCCTCGCGCCATCAGAGCACTGTAGTGTCAAGCAGTGCAACATTTC |
| EXITBPFUSAMID5 | CGTATCGCCTCCCTCGCGCCATCAGATCAGACACGTGTCAAGCAGTGCAACATTTC |
| EXITBPFUSAMID6 | CGTATCGCCTCCCTCGCGCCATCAGATATCGCGAGTGTCAAGCAGTGCAACATTTC |
| EXITBPFUSAMID7 | CGTATCGCCTCCCTCGCGCCATCAGCGTGTCTCTATGTCAAGCAGTGCAACATTTC |
| EXITBPFUSAMID8 | CGTATCGCCTCCCTCGCGCCATCAGCTCGCGTGTCTGTCAAGCAGTGCAACATTTC |
| EXITBPFUSAMID10 | CGTATCGCCTCCCTCGCGCCATCAGTCTCTATGCGTGTCAAGCAGTGCAACATTTC |
| EXITBPFUSAMID11 | CGTATCGCCTCCCTCGCGCCATCAGTGATACGTCTTGTCAAGCAGTGCAACATTTC |
| EXITBPFUSAMID13 | CGTATCGCCTCCCTCGCGCCATCAGCATAGTAGTGTGTCAAGCAGTGCAACATTTC |
| EXITBPFUSAMID14 | CGTATCGCCTCCCTCGCGCCATCAGCGAGAGATACTGTCAAGCAGTGCAACATTTC |
| EXITBPFUSAMID15 | CGTATCGCCTCCCTCGCGCCATCAGATACGACGTATGTCAAGCAGTGCAACATTTC |
| EXITBPFUSAMID16 | CGTATCGCCTCCCTCGCGCCATCAGTCACGTACTATGTCAAGCAGTGCAACATTTC |
| EXITBPFUSAMID17 | CGTATCGCCTCCCTCGCGCCATCAGCGTCTAGTACTGTCAAGCAGTGCAACATTTC |
| EXITBPFUSAMID18 | CGTATCGCCTCCCTCGCGCCATCAGTCTACGTAGCTGTCAAGCAGTGCAACATTTC |
| EXITBPFUSAMID19 | CGTATCGCCTCCCTCGCGCCATCAGTGTACTACTCTGTCAAGCAGTGCAACATTTC |
| EXITBPFUSAMID20 | CGTATCGCCTCCCTCGCGCCATCAGACGACTACAGTGTCAAGCAGTGCAACATTTC |
| EXITBPFUSAMID21 | CGTATCGCCTCCCTCGCGCCATCAGCGTAGACTAGTGTCAAGCAGTGCAACATTTC |
| EXITBPFUSAMID22 | CGTATCGCCTCCCTCGCGCCATCAGTACGAGTATGTGTCAAGCAGTGCAACATTTC |
| EXITBPFUSAMID23 | CGTATCGCCTCCCTCGCGCCATCAGTACTCTCGTGTGTCAAGCAGTGCAACATTTC |
| EXITBPFUSAMID24 | CGTATCGCCTCCCTCGCGCCATCAGTAGAGACGAGTGTCAAGCAGTGCAACATTTC |
| EXITBPFUSBMID1 | CTATGCGCCTTGCCAGCCCGCTCAGACGAGTGCGTGCTCCCTGATCCGCATAATA |
| EXITBPFUSBMID2 | CTATGCGCCTTGCCAGCCCGCTCAGACGCTCGACAGCTCCCTGATCCGCATAATA |
| EXITBPFUSBMID3 | CTATGCGCCTTGCCAGCCCGCTCAGAGACGCACTCGCTCCCTGATCCGCATAATA |
| EXITBPFUSBMID4 | CTATGCGCCTTGCCAGCCCGCTCAGAGCACTGTAGGCTCCCTGATCCGCATAATA |
| EXITBPFUSBMID5 | CTATGCGCCTTGCCAGCCCGCTCAGATCAGACACGGCTCCCTGATCCGCATAATA |
| EXITBPFUSBMID6 | CTATGCGCCTTGCCAGCCCGCTCAGATATCGCGAGGCTCCCTGATCCGCATAATA |
| EXITBPFUSBMID7 | CTATGCGCCTTGCCAGCCCGCTCAGCGTGTCTCTAGCTCCCTGATCCGCATAATA |
| EXITBPFUSBMID8 | CTATGCGCCTTGCCAGCCCGCTCAGCTCGCGTGTCGCTCCCTGATCCGCATAATA |
| EXITBPFUSBMID10 | CTATGCGCCTTGCCAGCCCGCTCAGTCTCTATGCGGCTCCCTGATCCGCATAATA |
| EXITBPFUSBMID11 | CTATGCGCCTTGCCAGCCCGCTCAGTGATACGTCTGCTCCCTGATCCGCATAATA |
| EXITBPFUSBMID13 | CTATGCGCCTTGCCAGCCCGCTCAGCATAGTAGTGGCTCCCTGATCCGCATAATA |
| EXITBPFUSBMID14 | CTATGCGCCTTGCCAGCCCGCTCAGCGAGAGATACGCTCCCTGATCCGCATAATA |
| EXITBPFUSBMID15 | CTATGCGCCTTGCCAGCCCGCTCAGATACGACGTAGCTCCCTGATCCGCATAATA |
| EXITBPFUSBMID16 | CTATGCGCCTTGCCAGCCCGCTCAGTCACGTACTAGCTCCCTGATCCGCATAATA |
| EXITBPFUSBMID17 | CTATGCGCCTTGCCAGCCCGCTCAGCGTCTAGTACGCTCCCTGATCCGCATAATA |
| EXITBPFUSBMID18 | CTATGCGCCTTGCCAGCCCGCTCAGTCTACGTAGCGCTCCCTGATCCGCATAATA |
| EXITBPFUSBMID19 | CTATGCGCCTTGCCAGCCCGCTCAGTGTACTACTCGCTCCCTGATCCGCATAATA |
| EXITBPFUSBMID20 | CTATGCGCCTTGCCAGCCCGCTCAGACGACTACAGGCTCCCTGATCCGCATAATA |
| EXITBPFUSBMID21 | CTATGCGCCTTGCCAGCCCGCTCAGCGTAGACTAGGCTCCCTGATCCGCATAATA |
| EXITBPFUSBMID22 | CTATGCGCCTTGCCAGCCCGCTCAGTACGAGTATGGCTCCCTGATCCGCATAATA |
| EXITBPFUSBMID23 | CTATGCGCCTTGCCAGCCCGCTCAGTACTCTCGTGGCTCCCTGATCCGCATAATA |
| EXITBPFUSBMID24 | CTATGCGCCTTGCCAGCCCGCTCAGTAGAGACGAGGCTCCCTGATCCGCATAATA |
| CALTBPFUSAMID1 | CGTATCGCCTCCCTCGCGCCATCAGACGAGTGCGTTGTTTCAAGCAATACAAAAAGGTT |
| CALTBPFUSAMID2 | CGTATCGCCTCCCTCGCGCCATCAGACGCTCGACATGTTTCAAGCAATACAAAAAGGTT |
| CALTBPFUSAMID3 | CGTATCGCCTCCCTCGCGCCATCAGAGACGCACTCTGTTTCAAGCAATACAAAAAGGTT |
| CALTBPFUSAMID4 | CGTATCGCCTCCCTCGCGCCATCAGAGCACTGTAGTGTTTCAAGCAATACAAAAAGGTT |
| CALTBPFUSAMID5 | CGTATCGCCTCCCTCGCGCCATCAGATCAGACACGTGTTTCAAGCAATACAAAAAGGTT |
| CALTBPFUSAMID6 | CGTATCGCCTCCCTCGCGCCATCAGATATCGCGAGTGTTTCAAGCAATACAAAAAGGTT |
| CALTBPFUSAMID7 | CGTATCGCCTCCCTCGCGCCATCAGCGTGTCTCTATGTTTCAAGCAATACAAAAAGGTT |
| CALTBPFUSAMID8 | CGTATCGCCTCCCTCGCGCCATCAGCTCGCGTGTCTGTTTCAAGCAATACAAAAAGGTT |
| CALTBPFUSAMID10 | CGTATCGCCTCCCTCGCGCCATCAGTCTCTATGCGTGTTTCAAGCAATACAAAAAGGTT |
| CALTBPFUSAMID11 | CGTATCGCCTCCCTCGCGCCATCAGTGATACGTCTTGTTTCAAGCAATACAAAAAGGTT |
| CALTBPFUSAMID13 | CGTATCGCCTCCCTCGCGCCATCAGCATAGTAGTGTGTTTCAAGCAATACAAAAAGGTT |
| CALTBPFUSAMID14 | CGTATCGCCTCCCTCGCGCCATCAGCGAGAGATACTGTTTCAAGCAATACAAAAAGGTT |
| CALTBPFUSAMID15 | CGTATCGCCTCCCTCGCGCCATCAGATACGACGTATGTTTCAAGCAATACAAAAAGGTT |
| CALTBPFUSAMID16 | CGTATCGCCTCCCTCGCGCCATCAGTCACGTACTATGTTTCAAGCAATACAAAAAGGTT |
| CALTBPFUSAMID17 | CGTATCGCCTCCCTCGCGCCATCAGCGTCTAGTACTGTTTCAAGCAATACAAAAAGGTT |
| CALTBPFUSAMID18 | CGTATCGCCTCCCTCGCGCCATCAGTCTACGTAGCTGTTTCAAGCAATACAAAAAGGTT |
| CALTBPFUSAMID19 | CGTATCGCCTCCCTCGCGCCATCAGTGTACTACTCTGTTTCAAGCAATACAAAAAGGTT |
| CALTBPFUSAMID20 | CGTATCGCCTCCCTCGCGCCATCAGACGACTACAGTGTTTCAAGCAATACAAAAAGGTT |
| CALTBPFUSAMID21 | CGTATCGCCTCCCTCGCGCCATCAGCGTAGACTAGTGTTTCAAGCAATACAAAAAGGTT |
| CALTBPFUSAMID22 | CGTATCGCCTCCCTCGCGCCATCAGTACGAGTATGTGTTTCAAGCAATACAAAAAGGTT |
| CALTBPFUSAMID23 | CGTATCGCCTCCCTCGCGCCATCAGTACTCTCGTGTGTTTCAAGCAATACAAAAAGGTT |
| CALTBPFUSAMID24 | CGTATCGCCTCCCTCGCGCCATCAGTAGAGACGAGTGTTTCAAGCAATACAAAAAGGTT |
| CALTBPFUSBMID1 | CTATGCGCCTTGCCAGCCCGCTCAGACGAGTGCGTGCACATTTGGCAACAGAAAT |
| CALTBPFUSBMID2 | CTATGCGCCTTGCCAGCCCGCTCAGACGCTCGACAGCACATTTGGCAACAGAAAT |
| CALTBPFUSBMID3 | CTATGCGCCTTGCCAGCCCGCTCAGAGACGCACTCGCACATTTGGCAACAGAAAT |
| CALTBPFUSBMID4 | CTATGCGCCTTGCCAGCCCGCTCAGAGCACTGTAGGCACATTTGGCAACAGAAAT |
| CALTBPFUSBMID5 | CTATGCGCCTTGCCAGCCCGCTCAGATCAGACACGGCACATTTGGCAACAGAAAT |
| CALTBPFUSBMID6 | CTATGCGCCTTGCCAGCCCGCTCAGATATCGCGAGGCACATTTGGCAACAGAAAT |
| CALTBPFUSBMID7 | CTATGCGCCTTGCCAGCCCGCTCAGCGTGTCTCTAGCACATTTGGCAACAGAAAT |
| CALTBPFUSBMID8 | CTATGCGCCTTGCCAGCCCGCTCAGCTCGCGTGTCGCACATTTGGCAACAGAAAT |
| CALTBPFUSBMID10 | CTATGCGCCTTGCCAGCCCGCTCAGTCTCTATGCGGCACATTTGGCAACAGAAAT |
| CALTBPFUSBMID11 | CTATGCGCCTTGCCAGCCCGCTCAGTGATACGTCTGCACATTTGGCAACAGAAAT |
| CALTBPFUSBMID13 | CTATGCGCCTTGCCAGCCCGCTCAGCATAGTAGTGGCACATTTGGCAACAGAAAT |
| CALTBPFUSBMID14 | CTATGCGCCTTGCCAGCCCGCTCAGCGAGAGATACGCACATTTGGCAACAGAAAT |
| CALTBPFUSBMID15 | CTATGCGCCTTGCCAGCCCGCTCAGATACGACGTAGCACATTTGGCAACAGAAAT |
| CALTBPFUSBMID16 | CTATGCGCCTTGCCAGCCCGCTCAGTCACGTACTAGCACATTTGGCAACAGAAAT |
| CALTBPFUSBMID17 | CTATGCGCCTTGCCAGCCCGCTCAGCGTCTAGTACGCACATTTGGCAACAGAAAT |
| CALTBPFUSBMID18 | CTATGCGCCTTGCCAGCCCGCTCAGTCTACGTAGCGCACATTTGGCAACAGAAAT |
| CALTBPFUSBMID19 | CTATGCGCCTTGCCAGCCCGCTCAGTGTACTACTCGCACATTTGGCAACAGAAAT |
| CALTBPFUSBMID20 | CTATGCGCCTTGCCAGCCCGCTCAGACGACTACAGGCACATTTGGCAACAGAAAT |
| CALTBPFUSBMID21 | CTATGCGCCTTGCCAGCCCGCTCAGCGTAGACTAGGCACATTTGGCAACAGAAAT |
| CALTBPFUSBMID22 | CTATGCGCCTTGCCAGCCCGCTCAGTACGAGTATGGCACATTTGGCAACAGAAAT |
| CALTBPFUSBMID23 | CTATGCGCCTTGCCAGCCCGCTCAGTACTCTCGTGGCACATTTGGCAACAGAAAT |
| CALTBPFUSBMID24 | CTATGCGCCTTGCCAGCCCGCTCAGTAGAGACGAGGCACATTTGGCAACAGAAAT |
| EXIATPSFUSAMID1 | CGTATCGCCTCCCTCGCGCCATCAGACGAGTGCGTTTTGCACCAGTGACCTTTTG |
| EXIATPSFUSAMID2 | CGTATCGCCTCCCTCGCGCCATCAGACGCTCGACATTTGCACCAGTGACCTTTTG |
| EXIATPSFUSAMID3 | CGTATCGCCTCCCTCGCGCCATCAGAGACGCACTCTTTGCACCAGTGACCTTTTG |
| EXIATPSFUSAMID4 | CGTATCGCCTCCCTCGCGCCATCAGAGCACTGTAGTTTGCACCAGTGACCTTTTG |
| EXIATPSFUSAMID5 | CGTATCGCCTCCCTCGCGCCATCAGATCAGACACGTTTGCACCAGTGACCTTTTG |
| EXIATPSFUSAMID6 | CGTATCGCCTCCCTCGCGCCATCAGATATCGCGAGTTTGCACCAGTGACCTTTTG |
| EXIATPSFUSAMID7 | CGTATCGCCTCCCTCGCGCCATCAGCGTGTCTCTATTTGCACCAGTGACCTTTTG |
| EXIATPSFUSAMID8 | CGTATCGCCTCCCTCGCGCCATCAGCTCGCGTGTCTTTGCACCAGTGACCTTTTG |
| EXIATPSFUSAMID10 | CGTATCGCCTCCCTCGCGCCATCAGTCTCTATGCGTTTGCACCAGTGACCTTTTG |
| EXIATPSFUSAMID11 | CGTATCGCCTCCCTCGCGCCATCAGTGATACGTCTTTTGCACCAGTGACCTTTTG |
| EXIATPSFUSAMID13 | CGTATCGCCTCCCTCGCGCCATCAGCATAGTAGTGTTTGCACCAGTGACCTTTTG |
| EXIATPSFUSAMID14 | CGTATCGCCTCCCTCGCGCCATCAGCGAGAGATACTTTGCACCAGTGACCTTTTG |
| EXIATPSFUSAMID15 | CGTATCGCCTCCCTCGCGCCATCAGATACGACGTATTTGCACCAGTGACCTTTTG |
| EXIATPSFUSAMID16 | CGTATCGCCTCCCTCGCGCCATCAGTCACGTACTATTTGCACCAGTGACCTTTTG |
| EXIATPSFUSAMID17 | CGTATCGCCTCCCTCGCGCCATCAGCGTCTAGTACTTTGCACCAGTGACCTTTTG |
| EXIATPSFUSAMID18 | CGTATCGCCTCCCTCGCGCCATCAGTCTACGTAGCTTTGCACCAGTGACCTTTTG |
| EXIATPSFUSAMID19 | CGTATCGCCTCCCTCGCGCCATCAGTGTACTACTCTTTGCACCAGTGACCTTTTG |
| EXIATPSFUSAMID20 | CGTATCGCCTCCCTCGCGCCATCAGACGACTACAGTTTGCACCAGTGACCTTTTG |
| EXIATPSFUSAMID21 | CGTATCGCCTCCCTCGCGCCATCAGCGTAGACTAGTTTGCACCAGTGACCTTTTG |
| EXIATPSFUSAMID22 | CGTATCGCCTCCCTCGCGCCATCAGTACGAGTATGTTTGCACCAGTGACCTTTTG |
| EXIATPSFUSAMID23 | CGTATCGCCTCCCTCGCGCCATCAGTACTCTCGTGTTTGCACCAGTGACCTTTTG |
| EXIATPSFUSAMID24 | CGTATCGCCTCCCTCGCGCCATCAGTAGAGACGAGTTTGCACCAGTGACCTTTTG |
| EXIATPSFUSBMID1 | CTATGCGCCTTGCCAGCCCGCTCAGACGAGTGCGTGCCCTTTGAGCTACAGTTCG |
| EXIATPSFUSBMID2 | CTATGCGCCTTGCCAGCCCGCTCAGACGCTCGACAGCCCTTTGAGCTACAGTTCG |
| EXIATPSFUSBMID3 | CTATGCGCCTTGCCAGCCCGCTCAGAGACGCACTCGCCCTTTGAGCTACAGTTCG |
| EXIATPSFUSBMID4 | CTATGCGCCTTGCCAGCCCGCTCAGAGCACTGTAGGCCCTTTGAGCTACAGTTCG |
| EXIATPSFUSBMID5 | CTATGCGCCTTGCCAGCCCGCTCAGATCAGACACGGCCCTTTGAGCTACAGTTCG |
| EXIATPSFUSBMID6 | CTATGCGCCTTGCCAGCCCGCTCAGATATCGCGAGGCCCTTTGAGCTACAGTTCG |
| EXIATPSFUSBMID7 | CTATGCGCCTTGCCAGCCCGCTCAGCGTGTCTCTAGCCCTTTGAGCTACAGTTCG |
| EXIATPSFUSBMID8 | CTATGCGCCTTGCCAGCCCGCTCAGCTCGCGTGTCGCCCTTTGAGCTACAGTTCG |
| EXIATPSFUSBMID10 | CTATGCGCCTTGCCAGCCCGCTCAGTCTCTATGCGGCCCTTTGAGCTACAGTTCG |
| EXIATPSFUSBMID11 | CTATGCGCCTTGCCAGCCCGCTCAGTGATACGTCTGCCCTTTGAGCTACAGTTCG |
| EXIATPSFUSBMID13 | CTATGCGCCTTGCCAGCCCGCTCAGCATAGTAGTGGCCCTTTGAGCTACAGTTCG |
| EXIATPSFUSBMID14 | CTATGCGCCTTGCCAGCCCGCTCAGCGAGAGATACGCCCTTTGAGCTACAGTTCG |
| EXIATPSFUSBMID15 | CTATGCGCCTTGCCAGCCCGCTCAGATACGACGTAGCCCTTTGAGCTACAGTTCG |
| EXIATPSFUSBMID16 | CTATGCGCCTTGCCAGCCCGCTCAGTCACGTACTAGCCCTTTGAGCTACAGTTCG |
| EXIATPSFUSBMID17 | CTATGCGCCTTGCCAGCCCGCTCAGCGTCTAGTACGCCCTTTGAGCTACAGTTCG |
| EXIATPSFUSBMID18 | CTATGCGCCTTGCCAGCCCGCTCAGTCTACGTAGCGCCCTTTGAGCTACAGTTCG |
| EXIATPSFUSBMID19 | CTATGCGCCTTGCCAGCCCGCTCAGTGTACTACTCGCCCTTTGAGCTACAGTTCG |
| EXIATPSFUSBMID20 | CTATGCGCCTTGCCAGCCCGCTCAGACGACTACAGGCCCTTTGAGCTACAGTTCG |
| EXIATPSFUSBMID21 | CTATGCGCCTTGCCAGCCCGCTCAGCGTAGACTAGGCCCTTTGAGCTACAGTTCG |
| EXIATPSFUSBMID22 | CTATGCGCCTTGCCAGCCCGCTCAGTACGAGTATGGCCCTTTGAGCTACAGTTCG |
| EXIATPSFUSBMID23 | CTATGCGCCTTGCCAGCCCGCTCAGTACTCTCGTGGCCCTTTGAGCTACAGTTCG |
| EXIATPSFUSBMID24 | CTATGCGCCTTGCCAGCCCGCTCAGTAGAGACGAGGCCCTTTGAGCTACAGTTCG |
| CALATPSFUSAMID1 | CGTATCGCCTCCCTCGCGCCATCAGACGAGTGCGTCGTGTCTTTTGCGGGTAAAC |
| CALATPSFUSAMID2 | CGTATCGCCTCCCTCGCGCCATCAGACGCTCGACACGTGTCTTTTGCGGGTAAAC |
| CALATPSFUSAMID3 | CGTATCGCCTCCCTCGCGCCATCAGAGACGCACTCCGTGTCTTTTGCGGGTAAAC |
| CALATPSFUSAMID4 | CGTATCGCCTCCCTCGCGCCATCAGAGCACTGTAGCGTGTCTTTTGCGGGTAAAC |
| CALATPSFUSAMID5 | CGTATCGCCTCCCTCGCGCCATCAGATCAGACACGCGTGTCTTTTGCGGGTAAAC |
| CALATPSFUSAMID6 | CGTATCGCCTCCCTCGCGCCATCAGATATCGCGAGCGTGTCTTTTGCGGGTAAAC |
| CALATPSFUSAMID7 | CGTATCGCCTCCCTCGCGCCATCAGCGTGTCTCTACGTGTCTTTTGCGGGTAAAC |
| CALATPSFUSAMID8 | CGTATCGCCTCCCTCGCGCCATCAGCTCGCGTGTCCGTGTCTTTTGCGGGTAAAC |
| CALATPSFUSAMID10 | CGTATCGCCTCCCTCGCGCCATCAGTCTCTATGCGCGTGTCTTTTGCGGGTAAAC |
| CALATPSFUSAMID11 | CGTATCGCCTCCCTCGCGCCATCAGTGATACGTCTCGTGTCTTTTGCGGGTAAAC |
| CALATPSFUSAMID13 | CGTATCGCCTCCCTCGCGCCATCAGCATAGTAGTGCGTGTCTTTTGCGGGTAAAC |
| CALATPSFUSAMID14 | CGTATCGCCTCCCTCGCGCCATCAGCGAGAGATACCGTGTCTTTTGCGGGTAAAC |
| CALATPSFUSAMID15 | CGTATCGCCTCCCTCGCGCCATCAGATACGACGTACGTGTCTTTTGCGGGTAAAC |
| CALATPSFUSAMID16 | CGTATCGCCTCCCTCGCGCCATCAGTCACGTACTACGTGTCTTTTGCGGGTAAAC |
| CALATPSFUSAMID17 | CGTATCGCCTCCCTCGCGCCATCAGCGTCTAGTACCGTGTCTTTTGCGGGTAAAC |
| CALATPSFUSAMID18 | CGTATCGCCTCCCTCGCGCCATCAGTCTACGTAGCCGTGTCTTTTGCGGGTAAAC |
| CALATPSFUSAMID19 | CGTATCGCCTCCCTCGCGCCATCAGTGTACTACTCCGTGTCTTTTGCGGGTAAAC |
| CALATPSFUSAMID20 | CGTATCGCCTCCCTCGCGCCATCAGACGACTACAGCGTGTCTTTTGCGGGTAAAC |
| CALATPSFUSAMID21 | CGTATCGCCTCCCTCGCGCCATCAGCGTAGACTAGCGTGTCTTTTGCGGGTAAAC |
| CALATPSFUSAMID22 | CGTATCGCCTCCCTCGCGCCATCAGTACGAGTATGCGTGTCTTTTGCGGGTAAAC |
| CALATPSFUSAMID23 | CGTATCGCCTCCCTCGCGCCATCAGTACTCTCGTGCGTGTCTTTTGCGGGTAAAC |
| CALATPSFUSAMID24 | CGTATCGCCTCCCTCGCGCCATCAGTAGAGACGAGCGTGTCTTTTGCGGGTAAAC |
| CALATPSFUSBMID1 | CTATGCGCCTTGCCAGCCCGCTCAGACGAGTGCGTAATGATGTTGGCACTTTTCG |
| CALATPSFUSBMID2 | CTATGCGCCTTGCCAGCCCGCTCAGACGCTCGACAAATGATGTTGGCACTTTTCG |
| CALATPSFUSBMID3 | CTATGCGCCTTGCCAGCCCGCTCAGAGACGCACTCAATGATGTTGGCACTTTTCG |
| CALATPSFUSBMID4 | CTATGCGCCTTGCCAGCCCGCTCAGAGCACTGTAGAATGATGTTGGCACTTTTCG |
| CALATPSFUSBMID5 | CTATGCGCCTTGCCAGCCCGCTCAGATCAGACACGAATGATGTTGGCACTTTTCG |
| CALATPSFUSBMID6 | CTATGCGCCTTGCCAGCCCGCTCAGATATCGCGAGAATGATGTTGGCACTTTTCG |
| CALATPSFUSBMID7 | CTATGCGCCTTGCCAGCCCGCTCAGCGTGTCTCTAAATGATGTTGGCACTTTTCG |
| CALATPSFUSBMID8 | CTATGCGCCTTGCCAGCCCGCTCAGCTCGCGTGTCAATGATGTTGGCACTTTTCG |
| CALATPSFUSBMID10 | CTATGCGCCTTGCCAGCCCGCTCAGTCTCTATGCGAATGATGTTGGCACTTTTCG |
| CALATPSFUSBMID11 | CTATGCGCCTTGCCAGCCCGCTCAGTGATACGTCTAATGATGTTGGCACTTTTCG |
| CALATPSFUSBMID13 | CTATGCGCCTTGCCAGCCCGCTCAGCATAGTAGTGAATGATGTTGGCACTTTTCG |
| CALATPSFUSBMID14 | CTATGCGCCTTGCCAGCCCGCTCAGCGAGAGATACAATGATGTTGGCACTTTTCG |
| CALATPSFUSBMID15 | CTATGCGCCTTGCCAGCCCGCTCAGATACGACGTAAATGATGTTGGCACTTTTCG |
| CALATPSFUSBMID16 | CTATGCGCCTTGCCAGCCCGCTCAGTCACGTACTAAATGATGTTGGCACTTTTCG |
| CALATPSFUSBMID17 | CTATGCGCCTTGCCAGCCCGCTCAGCGTCTAGTACAATGATGTTGGCACTTTTCG |
| CALATPSFUSBMID18 | CTATGCGCCTTGCCAGCCCGCTCAGTCTACGTAGCAATGATGTTGGCACTTTTCG |
| CALATPSFUSBMID19 | CTATGCGCCTTGCCAGCCCGCTCAGTGTACTACTCAATGATGTTGGCACTTTTCG |
| CALATPSFUSBMID20 | CTATGCGCCTTGCCAGCCCGCTCAGACGACTACAGAATGATGTTGGCACTTTTCG |
| CALATPSFUSBMID21 | CTATGCGCCTTGCCAGCCCGCTCAGCGTAGACTAGAATGATGTTGGCACTTTTCG |
| CALATPSFUSBMID22 | CTATGCGCCTTGCCAGCCCGCTCAGTACGAGTATGAATGATGTTGGCACTTTTCG |
| CALATPSFUSBMID23 | CTATGCGCCTTGCCAGCCCGCTCAGTACTCTCGTGAATGATGTTGGCACTTTTCG |
| CALATPSFUSBMID24 | CTATGCGCCTTGCCAGCCCGCTCAGTAGAGACGAGAATGATGTTGGCACTTTTCG |
| CALEFAFUSAMID1 | CGTATCGCCTCCCTCGCGCCATCAGACGAGTGCGTAGTTAACCCAGTCACCAAGAGTCAWA |
| CALEFAFUSAMID2 | CGTATCGCCTCCCTCGCGCCATCAGACGCTCGACAAGTTAACCCAGTCACCAAGAGTCAWA |
| CALEFAFUSAMID3 | CGTATCGCCTCCCTCGCGCCATCAGAGACGCACTCAGTTAACCCAGTCACCAAGAGTCAWA |
| CALEFAFUSAMID4 | CGTATCGCCTCCCTCGCGCCATCAGAGCACTGTAGAGTTAACCCAGTCACCAAGAGTCAWA |
| CALEFAFUSAMID5 | CGTATCGCCTCCCTCGCGCCATCAGATCAGACACGAGTTAACCCAGTCACCAAGAGTCAWA |
| CALEFAFUSAMID6 | CGTATCGCCTCCCTCGCGCCATCAGATATCGCGAGAGTTAACCCAGTCACCAAGAGTCAWA |
| CALEFAFUSAMID7 | CGTATCGCCTCCCTCGCGCCATCAGCGTGTCTCTAAGTTAACCCAGTCACCAAGAGTCAWA |
| CALEFAFUSAMID8 | CGTATCGCCTCCCTCGCGCCATCAGCTCGCGTGTCAGTTAACCCAGTCACCAAGAGTCAWA |
| CALEFAFUSAMID10 | CGTATCGCCTCCCTCGCGCCATCAGTCTCTATGCGAGTTAACCCAGTCACCAAGAGTCAWA |
| CALEFAFUSAMID11 | CGTATCGCCTCCCTCGCGCCATCAGTGATACGTCTAGTTAACCCAGTCACCAAGAGTCAWA |
| CALEFAFUSAMID13 | CGTATCGCCTCCCTCGCGCCATCAGCATAGTAGTGAGTTAACCCAGTCACCAAGAGTCAWA |
| CALEFAFUSAMID14 | CGTATCGCCTCCCTCGCGCCATCAGCGAGAGATACAGTTAACCCAGTCACCAAGAGTCAWA |
| CALEFAFUSAMID15 | CGTATCGCCTCCCTCGCGCCATCAGATACGACGTAAGTTAACCCAGTCACCAAGAGTCAWA |
| CALEFAFUSAMID16 | CGTATCGCCTCCCTCGCGCCATCAGTCACGTACTAAGTTAACCCAGTCACCAAGAGTCAWA |
| CALEFAFUSAMID17 | CGTATCGCCTCCCTCGCGCCATCAGCGTCTAGTACAGTTAACCCAGTCACCAAGAGTCAWA |
| CALEFAFUSAMID18 | CGTATCGCCTCCCTCGCGCCATCAGTCTACGTAGCAGTTAACCCAGTCACCAAGAGTCAWA |
| CALEFAFUSAMID19 | CGTATCGCCTCCCTCGCGCCATCAGTGTACTACTCAGTTAACCCAGTCACCAAGAGTCAWA |
| CALEFAFUSAMID20 | CGTATCGCCTCCCTCGCGCCATCAGACGACTACAGAGTTAACCCAGTCACCAAGAGTCAWA |
| CALEFAFUSAMID21 | CGTATCGCCTCCCTCGCGCCATCAGCGTAGACTAGAGTTAACCCAGTCACCAAGAGTCAWA |
| CALEFAFUSAMID22 | CGTATCGCCTCCCTCGCGCCATCAGTACGAGTATGAGTTAACCCAGTCACCAAGAGTCAWA |
| CALEFAFUSAMID23 | CGTATCGCCTCCCTCGCGCCATCAGTACTCTCGTGAGTTAACCCAGTCACCAAGAGTCAWA |
| CALEFAFUSAMID24 | CGTATCGCCTCCCTCGCGCCATCAGTAGAGACGAGAGTTAACCCAGTCACCAAGAGTCAWA |
| CALEFAFUSBMID1 | CTATGCGCCTTGCCAGCCCGCTCAGACGAGTGCGTGGACCAAGTAGAAGGATTGCCCTC |
| CALEFAFUSBMID2 | CTATGCGCCTTGCCAGCCCGCTCAGACGCTCGACAGGACCAAGTAGAAGGATTGCCCTC |
| CALEFAFUSBMID3 | CTATGCGCCTTGCCAGCCCGCTCAGAGACGCACTCGGACCAAGTAGAAGGATTGCCCTC |
| CALEFAFUSBMID4 | CTATGCGCCTTGCCAGCCCGCTCAGAGCACTGTAGGGACCAAGTAGAAGGATTGCCCTC |
| CALEFAFUSBMID5 | CTATGCGCCTTGCCAGCCCGCTCAGATCAGACACGGGACCAAGTAGAAGGATTGCCCTC |
| CALEFAFUSBMID6 | CTATGCGCCTTGCCAGCCCGCTCAGATATCGCGAGGGACCAAGTAGAAGGATTGCCCTC |
| CALEFAFUSBMID7 | CTATGCGCCTTGCCAGCCCGCTCAGCGTGTCTCTAGGACCAAGTAGAAGGATTGCCCTC |
| CALEFAFUSBMID8 | CTATGCGCCTTGCCAGCCCGCTCAGCTCGCGTGTCGGACCAAGTAGAAGGATTGCCCTC |
| CALEFAFUSBMID10 | CTATGCGCCTTGCCAGCCCGCTCAGTCTCTATGCGGGACCAAGTAGAAGGATTGCCCTC |
| CALEFAFUSBMID11 | CTATGCGCCTTGCCAGCCCGCTCAGTGATACGTCTGGACCAAGTAGAAGGATTGCCCTC |
| CALEFAFUSBMID13 | CTATGCGCCTTGCCAGCCCGCTCAGCATAGTAGTGGGACCAAGTAGAAGGATTGCCCTC |
| CALEFAFUSBMID14 | CTATGCGCCTTGCCAGCCCGCTCAGCGAGAGATACGGACCAAGTAGAAGGATTGCCCTC |
| CALEFAFUSBMID15 | CTATGCGCCTTGCCAGCCCGCTCAGATACGACGTAGGACCAAGTAGAAGGATTGCCCTC |
| CALEFAFUSBMID16 | CTATGCGCCTTGCCAGCCCGCTCAGTCACGTACTAGGACCAAGTAGAAGGATTGCCCTC |
| CALEFAFUSBMID17 | CTATGCGCCTTGCCAGCCCGCTCAGCGTCTAGTACGGACCAAGTAGAAGGATTGCCCTC |
| CALEFAFUSBMID18 | CTATGCGCCTTGCCAGCCCGCTCAGTCTACGTAGCGGACCAAGTAGAAGGATTGCCCTC |
| CALEFAFUSBMID19 | CTATGCGCCTTGCCAGCCCGCTCAGTGTACTACTCGGACCAAGTAGAAGGATTGCCCTC |
| CALEFAFUSBMID20 | CTATGCGCCTTGCCAGCCCGCTCAGACGACTACAGGGACCAAGTAGAAGGATTGCCCTC |
| CALEFAFUSBMID21 | CTATGCGCCTTGCCAGCCCGCTCAGCGTAGACTAGGGACCAAGTAGAAGGATTGCCCTC |
| CALEFAFUSBMID22 | CTATGCGCCTTGCCAGCCCGCTCAGTACGAGTATGGGACCAAGTAGAAGGATTGCCCTC |
| CALEFAFUSBMID23 | CTATGCGCCTTGCCAGCCCGCTCAGTACTCTCGTGGGACCAAGTAGAAGGATTGCCCTC |
| CALEFAFUSBMID24 | CTATGCGCCTTGCCAGCCCGCTCAGTAGAGACGAGGGACCAAGTAGAAGGATTGCCCTC |
| EXIEFAFUSAMID1 | CGTATCGCCTCCCTCGCGCCATCAGACGAGTGCGTAAAGGAAGCCGCTGAGGTGAGT |
| EXIEFAFUSAMID2 | CGTATCGCCTCCCTCGCGCCATCAGACGCTCGACAAAAGGAAGCCGCTGAGGTGAGT |
| EXIEFAFUSAMID3 | CGTATCGCCTCCCTCGCGCCATCAGAGACGCACTCAAAGGAAGCCGCTGAGGTGAGT |
| EXIEFAFUSAMID4 | CGTATCGCCTCCCTCGCGCCATCAGAGCACTGTAGAAAGGAAGCCGCTGAGGTGAGT |
| EXIEFAFUSAMID5 | CGTATCGCCTCCCTCGCGCCATCAGATCAGACACGAAAGGAAGCCGCTGAGGTGAGT |
| EXIEFAFUSAMID6 | CGTATCGCCTCCCTCGCGCCATCAGATATCGCGAGAAAGGAAGCCGCTGAGGTGAGT |
| EXIEFAFUSAMID7 | CGTATCGCCTCCCTCGCGCCATCAGCGTGTCTCTAAAAGGAAGCCGCTGAGGTGAGT |
| EXIEFAFUSAMID8 | CGTATCGCCTCCCTCGCGCCATCAGCTCGCGTGTCAAAGGAAGCCGCTGAGGTGAGT |
| EXIEFAFUSAMID10 | CGTATCGCCTCCCTCGCGCCATCAGTCTCTATGCGAAAGGAAGCCGCTGAGGTGAGT |
| EXIEFAFUSAMID11 | CGTATCGCCTCCCTCGCGCCATCAGTGATACGTCTAAAGGAAGCCGCTGAGGTGAGT |
| EXIEFAFUSAMID13 | CGTATCGCCTCCCTCGCGCCATCAGCATAGTAGTGAAAGGAAGCCGCTGAGGTGAGT |
| EXIEFAFUSAMID14 | CGTATCGCCTCCCTCGCGCCATCAGCGAGAGATACAAAGGAAGCCGCTGAGGTGAGT |
| EXIEFAFUSAMID15 | CGTATCGCCTCCCTCGCGCCATCAGATACGACGTAAAAGGAAGCCGCTGAGGTGAGT |
| EXIEFAFUSAMID16 | CGTATCGCCTCCCTCGCGCCATCAGTCACGTACTAAAAGGAAGCCGCTGAGGTGAGT |
| EXIEFAFUSAMID17 | CGTATCGCCTCCCTCGCGCCATCAGCGTCTAGTACAAAGGAAGCCGCTGAGGTGAGT |
| EXIEFAFUSAMID18 | CGTATCGCCTCCCTCGCGCCATCAGTCTACGTAGCAAAGGAAGCCGCTGAGGTGAGT |
| EXIEFAFUSAMID19 | CGTATCGCCTCCCTCGCGCCATCAGTGTACTACTCAAAGGAAGCCGCTGAGGTGAGT |
| EXIEFAFUSAMID20 | CGTATCGCCTCCCTCGCGCCATCAGACGACTACAGAAAGGAAGCCGCTGAGGTGAGT |
| EXIEFAFUSAMID21 | CGTATCGCCTCCCTCGCGCCATCAGCGTAGACTAGAAAGGAAGCCGCTGAGGTGAGT |
| EXIEFAFUSAMID22 | CGTATCGCCTCCCTCGCGCCATCAGTACGAGTATGAAAGGAAGCCGCTGAGGTGAGT |
| EXIEFAFUSAMID23 | CGTATCGCCTCCCTCGCGCCATCAGTACTCTCGTGAAAGGAAGCCGCTGAGGTGAGT |
| EXIEFAFUSAMID24 | CGTATCGCCTCCCTCGCGCCATCAGTAGAGACGAGAAAGGAAGCCGCTGAGGTGAGT |
| EXIEFAFUSBMID1 | CTATGCGCCTTGCCAGCCCGCTCAGACGAGTGCGTCGATGACGGTGCAGTAATACC |
| EXIEFAFUSBMID2 | CTATGCGCCTTGCCAGCCCGCTCAGACGCTCGACACGATGACGGTGCAGTAATACC |
| EXIEFAFUSBMID3 | CTATGCGCCTTGCCAGCCCGCTCAGAGACGCACTCCGATGACGGTGCAGTAATACC |
| EXIEFAFUSBMID4 | CTATGCGCCTTGCCAGCCCGCTCAGAGCACTGTAGCGATGACGGTGCAGTAATACC |
| EXIEFAFUSBMID5 | CTATGCGCCTTGCCAGCCCGCTCAGATCAGACACGCGATGACGGTGCAGTAATACC |
| EXIEFAFUSBMID6 | CTATGCGCCTTGCCAGCCCGCTCAGATATCGCGAGCGATGACGGTGCAGTAATACC |
| EXIEFAFUSBMID7 | CTATGCGCCTTGCCAGCCCGCTCAGCGTGTCTCTACGATGACGGTGCAGTAATACC |
| EXIEFAFUSBMID8 | CTATGCGCCTTGCCAGCCCGCTCAGCTCGCGTGTCCGATGACGGTGCAGTAATACC |
| EXIEFAFUSBMID10 | CTATGCGCCTTGCCAGCCCGCTCAGTCTCTATGCGCGATGACGGTGCAGTAATACC |
| EXIEFAFUSBMID11 | CTATGCGCCTTGCCAGCCCGCTCAGTGATACGTCTCGATGACGGTGCAGTAATACC |
| EXIEFAFUSBMID13 | CTATGCGCCTTGCCAGCCCGCTCAGCATAGTAGTGCGATGACGGTGCAGTAATACC |
| EXIEFAFUSBMID14 | CTATGCGCCTTGCCAGCCCGCTCAGCGAGAGATACCGATGACGGTGCAGTAATACC |
| EXIEFAFUSBMID15 | CTATGCGCCTTGCCAGCCCGCTCAGATACGACGTACGATGACGGTGCAGTAATACC |
| EXIEFAFUSBMID16 | CTATGCGCCTTGCCAGCCCGCTCAGTCACGTACTACGATGACGGTGCAGTAATACC |
| EXIEFAFUSBMID17 | CTATGCGCCTTGCCAGCCCGCTCAGCGTCTAGTACCGATGACGGTGCAGTAATACC |
| EXIEFAFUSBMID18 | CTATGCGCCTTGCCAGCCCGCTCAGTCTACGTAGCCGATGACGGTGCAGTAATACC |
| EXIEFAFUSBMID19 | CTATGCGCCTTGCCAGCCCGCTCAGTGTACTACTCCGATGACGGTGCAGTAATACC |
| EXIEFAFUSBMID20 | CTATGCGCCTTGCCAGCCCGCTCAGACGACTACAGCGATGACGGTGCAGTAATACC |
| EXIEFAFUSBMID21 | CTATGCGCCTTGCCAGCCCGCTCAGCGTAGACTAGCGATGACGGTGCAGTAATACC |
| EXIEFAFUSBMID22 | CTATGCGCCTTGCCAGCCCGCTCAGTACGAGTATGCGATGACGGTGCAGTAATACC |
| EXIEFAFUSBMID23 | CTATGCGCCTTGCCAGCCCGCTCAGTACTCTCGTGCGATGACGGTGCAGTAATACC |
| EXIEFAFUSBMID24 | CTATGCGCCTTGCCAGCCCGCTCAGTAGAGACGAGCGATGACGGTGCAGTAATACC |
| EXIGPIFUSAMID1 | CGTATCGCCTCCCTCGCGCCATCAGACGAGTGCGTGCCAAGCACTTTGTTGCCTT |
| EXIGPIFUSAMID2 | CGTATCGCCTCCCTCGCGCCATCAGACGCTCGACAGCCAAGCACTTTGTTGCCTT |
| EXIGPIFUSAMID3 | CGTATCGCCTCCCTCGCGCCATCAGAGACGCACTCGCCAAGCACTTTGTTGCCTT |
| EXIGPIFUSAMID4 | CGTATCGCCTCCCTCGCGCCATCAGAGCACTGTAGGCCAAGCACTTTGTTGCCTT |
| EXIGPIFUSAMID5 | CGTATCGCCTCCCTCGCGCCATCAGATCAGACACGGCCAAGCACTTTGTTGCCTT |
| EXIGPIFUSAMID6 | CGTATCGCCTCCCTCGCGCCATCAGATATCGCGAGGCCAAGCACTTTGTTGCCTT |
| EXIGPIFUSAMID7 | CGTATCGCCTCCCTCGCGCCATCAGCGTGTCTCTAGCCAAGCACTTTGTTGCCTT |
| EXIGPIFUSAMID8 | CGTATCGCCTCCCTCGCGCCATCAGCTCGCGTGTCGCCAAGCACTTTGTTGCCTT |
| EXIGPIFUSAMID10 | CGTATCGCCTCCCTCGCGCCATCAGTCTCTATGCGGCCAAGCACTTTGTTGCCTT |
| EXIGPIFUSAMID11 | CGTATCGCCTCCCTCGCGCCATCAGTGATACGTCTGCCAAGCACTTTGTTGCCTT |
| EXIGPIFUSAMID13 | CGTATCGCCTCCCTCGCGCCATCAGCATAGTAGTGGCCAAGCACTTTGTTGCCTT |
| EXIGPIFUSAMID14 | CGTATCGCCTCCCTCGCGCCATCAGCGAGAGATACGCCAAGCACTTTGTTGCCTT |
| EXIGPIFUSAMID15 | CGTATCGCCTCCCTCGCGCCATCAGATACGACGTAGCCAAGCACTTTGTTGCCTT |
| EXIGPIFUSAMID16 | CGTATCGCCTCCCTCGCGCCATCAGTCACGTACTAGCCAAGCACTTTGTTGCCTT |
| EXIGPIFUSAMID17 | CGTATCGCCTCCCTCGCGCCATCAGCGTCTAGTACGCCAAGCACTTTGTTGCCTT |
| EXIGPIFUSAMID18 | CGTATCGCCTCCCTCGCGCCATCAGTCTACGTAGCGCCAAGCACTTTGTTGCCTT |
| EXIGPIFUSAMID19 | CGTATCGCCTCCCTCGCGCCATCAGTGTACTACTCGCCAAGCACTTTGTTGCCTT |
| EXIGPIFUSAMID20 | CGTATCGCCTCCCTCGCGCCATCAGACGACTACAGGCCAAGCACTTTGTTGCCTT |
| EXIGPIFUSAMID21 | CGTATCGCCTCCCTCGCGCCATCAGCGTAGACTAGGCCAAGCACTTTGTTGCCTT |
| EXIGPIFUSAMID22 | CGTATCGCCTCCCTCGCGCCATCAGTACGAGTATGGCCAAGCACTTTGTTGCCTT |
| EXIGPIFUSAMID23 | CGTATCGCCTCCCTCGCGCCATCAGTACTCTCGTGGCCAAGCACTTTGTTGCCTT |
| EXIGPIFUSAMID24 | CGTATCGCCTCCCTCGCGCCATCAGTAGAGACGAGGCCAAGCACTTTGTTGCCTT |
| EXIGPIFUSBMID1 | CTATGCGCCTTGCCAGCCCGCTCAGACGAGTGCGTTCCCAGAAGGGAAACATGTTATCCTTGTCG |
| EXIGPIFUSBMID2 | CTATGCGCCTTGCCAGCCCGCTCAGACGCTCGACATCCCAGAAGGGAAACATGTTATCCTTGTCG |
| EXIGPIFUSBMID3 | CTATGCGCCTTGCCAGCCCGCTCAGAGACGCACTCTCCCAGAAGGGAAACATGTTATCCTTGTCG |
| EXIGPIFUSBMID4 | CTATGCGCCTTGCCAGCCCGCTCAGAGCACTGTAGTCCCAGAAGGGAAACATGTTATCCTTGTCG |
| EXIGPIFUSBMID5 | CTATGCGCCTTGCCAGCCCGCTCAGATCAGACACGTCCCAGAAGGGAAACATGTTATCCTTGTCG |
| EXIGPIFUSBMID6 | CTATGCGCCTTGCCAGCCCGCTCAGATATCGCGAGTCCCAGAAGGGAAACATGTTATCCTTGTCG |
| EXIGPIFUSBMID7 | CTATGCGCCTTGCCAGCCCGCTCAGCGTGTCTCTATCCCAGAAGGGAAACATGTTATCCTTGTCG |
| EXIGPIFUSBMID8 | CTATGCGCCTTGCCAGCCCGCTCAGCTCGCGTGTCTCCCAGAAGGGAAACATGTTATCCTTGTCG |
| EXIGPIFUSBMID10 | CTATGCGCCTTGCCAGCCCGCTCAGTCTCTATGCGTCCCAGAAGGGAAACATGTTATCCTTGTCG |
| EXIGPIFUSBMID11 | CTATGCGCCTTGCCAGCCCGCTCAGTGATACGTCTTCCCAGAAGGGAAACATGTTATCCTTGTCG |
| EXIGPIFUSBMID13 | CTATGCGCCTTGCCAGCCCGCTCAGCATAGTAGTGTCCCAGAAGGGAAACATGTTATCCTTGTCG |
| EXIGPIFUSBMID14 | CTATGCGCCTTGCCAGCCCGCTCAGCGAGAGATACTCCCAGAAGGGAAACATGTTATCCTTGTCG |
| EXIGPIFUSBMID15 | CTATGCGCCTTGCCAGCCCGCTCAGATACGACGTATCCCAGAAGGGAAACATGTTATCCTTGTCG |
| EXIGPIFUSBMID16 | CTATGCGCCTTGCCAGCCCGCTCAGTCACGTACTATCCCAGAAGGGAAACATGTTATCCTTGTCG |
| EXIGPIFUSBMID17 | CTATGCGCCTTGCCAGCCCGCTCAGCGTCTAGTACTCCCAGAAGGGAAACATGTTATCCTTGTCG |
| EXIGPIFUSBMID18 | CTATGCGCCTTGCCAGCCCGCTCAGTCTACGTAGCTCCCAGAAGGGAAACATGTTATCCTTGTCG |
| EXIGPIFUSBMID19 | CTATGCGCCTTGCCAGCCCGCTCAGTGTACTACTCTCCCAGAAGGGAAACATGTTATCCTTGTCG |
| EXIGPIFUSBMID20 | CTATGCGCCTTGCCAGCCCGCTCAGACGACTACAGTCCCAGAAGGGAAACATGTTATCCTTGTCG |
| EXIGPIFUSBMID21 | CTATGCGCCTTGCCAGCCCGCTCAGCGTAGACTAGTCCCAGAAGGGAAACATGTTATCCTTGTCG |
| EXIGPIFUSBMID22 | CTATGCGCCTTGCCAGCCCGCTCAGTACGAGTATGTCCCAGAAGGGAAACATGTTATCCTTGTCG |
| EXIGPIFUSBMID23 | CTATGCGCCTTGCCAGCCCGCTCAGTACTCTCGTGTCCCAGAAGGGAAACATGTTATCCTTGTCG |
| EXIGPIFUSBMID24 | CTATGCGCCTTGCCAGCCCGCTCAGTAGAGACGAGTCCCAGAAGGGAAACATGTTATCCTTGTCG |
| CALGPIFUSAMID1 | CGTATCGCCTCCCTCGCGCCATCAGACGAGTGCGTGTGGCCCTGTCAACCAACG |
| CALGPIFUSAMID2 | CGTATCGCCTCCCTCGCGCCATCAGACGCTCGACAGTGGCCCTGTCAACCAACG |
| CALGPIFUSAMID3 | CGTATCGCCTCCCTCGCGCCATCAGAGACGCACTCGTGGCCCTGTCAACCAACG |
| CALGPIFUSAMID4 | CGTATCGCCTCCCTCGCGCCATCAGAGCACTGTAGGTGGCCCTGTCAACCAACG |
| CALGPIFUSAMID5 | CGTATCGCCTCCCTCGCGCCATCAGATCAGACACGGTGGCCCTGTCAACCAACG |
| CALGPIFUSAMID6 | CGTATCGCCTCCCTCGCGCCATCAGATATCGCGAGGTGGCCCTGTCAACCAACG |
| CALGPIFUSAMID7 | CGTATCGCCTCCCTCGCGCCATCAGCGTGTCTCTAGTGGCCCTGTCAACCAACG |
| CALGPIFUSAMID8 | CGTATCGCCTCCCTCGCGCCATCAGCTCGCGTGTCGTGGCCCTGTCAACCAACG |
| CALGPIFUSAMID10 | CGTATCGCCTCCCTCGCGCCATCAGTCTCTATGCGGTGGCCCTGTCAACCAACG |
| CALGPIFUSAMID11 | CGTATCGCCTCCCTCGCGCCATCAGTGATACGTCTGTGGCCCTGTCAACCAACG |
| CALGPIFUSAMID13 | CGTATCGCCTCCCTCGCGCCATCAGCATAGTAGTGGTGGCCCTGTCAACCAACG |
| CALGPIFUSAMID14 | CGTATCGCCTCCCTCGCGCCATCAGCGAGAGATACGTGGCCCTGTCAACCAACG |
| CALGPIFUSAMID15 | CGTATCGCCTCCCTCGCGCCATCAGATACGACGTAGTGGCCCTGTCAACCAACG |
| CALGPIFUSAMID16 | CGTATCGCCTCCCTCGCGCCATCAGTCACGTACTAGTGGCCCTGTCAACCAACG |
| CALGPIFUSAMID17 | CGTATCGCCTCCCTCGCGCCATCAGCGTCTAGTACGTGGCCCTGTCAACCAACG |
| CALGPIFUSAMID18 | CGTATCGCCTCCCTCGCGCCATCAGTCTACGTAGCGTGGCCCTGTCAACCAACG |
| CALGPIFUSAMID19 | CGTATCGCCTCCCTCGCGCCATCAGTGTACTACTCGTGGCCCTGTCAACCAACG |
| CALGPIFUSAMID20 | CGTATCGCCTCCCTCGCGCCATCAGACGACTACAGGTGGCCCTGTCAACCAACG |
| CALGPIFUSAMID21 | CGTATCGCCTCCCTCGCGCCATCAGCGTAGACTAGGTGGCCCTGTCAACCAACG |
| CALGPIFUSAMID22 | CGTATCGCCTCCCTCGCGCCATCAGTACGAGTATGGTGGCCCTGTCAACCAACG |
| CALGPIFUSAMID23 | CGTATCGCCTCCCTCGCGCCATCAGTACTCTCGTGGTGGCCCTGTCAACCAACG |
| CALGPIFUSAMID24 | CGTATCGCCTCCCTCGCGCCATCAGTAGAGACGAGGTGGCCCTGTCAACCAACG |
| CALGPIFUSBMID1 | CTATGCGCCTTGCCAGCCCGCTCAGACGAGTGCGTTCCCARAARGGAAACATGTTWTCC |
| CALGPIFUSBMID2 | CTATGCGCCTTGCCAGCCCGCTCAGACGCTCGACATCCCARAARGGAAACATGTTWTCC |
| CALGPIFUSBMID3 | CTATGCGCCTTGCCAGCCCGCTCAGAGACGCACTCTCCCARAARGGAAACATGTTWTCC |
| CALGPIFUSBMID4 | CTATGCGCCTTGCCAGCCCGCTCAGAGCACTGTAGTCCCARAARGGAAACATGTTWTCC |
| CALGPIFUSBMID5 | CTATGCGCCTTGCCAGCCCGCTCAGATCAGACACGTCCCARAARGGAAACATGTTWTCC |
| CALGPIFUSBMID6 | CTATGCGCCTTGCCAGCCCGCTCAGATATCGCGAGTCCCARAARGGAAACATGTTWTCC |
| CALGPIFUSBMID7 | CTATGCGCCTTGCCAGCCCGCTCAGCGTGTCTCTATCCCARAARGGAAACATGTTWTCC |
| CALGPIFUSBMID8 | CTATGCGCCTTGCCAGCCCGCTCAGCTCGCGTGTCTCCCARAARGGAAACATGTTWTCC |
| CALGPIFUSBMID10 | CTATGCGCCTTGCCAGCCCGCTCAGTCTCTATGCGTCCCARAARGGAAACATGTTWTCC |
| CALGPIFUSBMID11 | CTATGCGCCTTGCCAGCCCGCTCAGTGATACGTCTTCCCARAARGGAAACATGTTWTCC |
| CALGPIFUSBMID13 | CTATGCGCCTTGCCAGCCCGCTCAGCATAGTAGTGTCCCARAARGGAAACATGTTWTCC |
| CALGPIFUSBMID14 | CTATGCGCCTTGCCAGCCCGCTCAGCGAGAGATACTCCCARAARGGAAACATGTTWTCC |
| CALGPIFUSBMID15 | CTATGCGCCTTGCCAGCCCGCTCAGATACGACGTATCCCARAARGGAAACATGTTWTCC |
| CALGPIFUSBMID16 | CTATGCGCCTTGCCAGCCCGCTCAGTCACGTACTATCCCARAARGGAAACATGTTWTCC |
| CALGPIFUSBMID17 | CTATGCGCCTTGCCAGCCCGCTCAGCGTCTAGTACTCCCARAARGGAAACATGTTWTCC |
| CALGPIFUSBMID18 | CTATGCGCCTTGCCAGCCCGCTCAGTCTACGTAGCTCCCARAARGGAAACATGTTWTCC |
| CALGPIFUSBMID19 | CTATGCGCCTTGCCAGCCCGCTCAGTGTACTACTCTCCCARAARGGAAACATGTTWTCC |
| CALGPIFUSBMID20 | CTATGCGCCTTGCCAGCCCGCTCAGACGACTACAGTCCCARAARGGAAACATGTTWTCC |
| CALGPIFUSBMID21 | CTATGCGCCTTGCCAGCCCGCTCAGCGTAGACTAGTCCCARAARGGAAACATGTTWTCC |
| CALGPIFUSBMID22 | CTATGCGCCTTGCCAGCCCGCTCAGTACGAGTATGTCCCARAARGGAAACATGTTWTCC |
| CALGPIFUSBMID23 | CTATGCGCCTTGCCAGCCCGCTCAGTACTCTCGTGTCCCARAARGGAAACATGTTWTCC |
| CALGPIFUSBMID24 | CTATGCGCCTTGCCAGCCCGCTCAGTAGAGACGAGTCCCARAARGGAAACATGTTWTCC |
